# MdMPK6a/b‐mediated phosphorylation of MdANN2 restricts cytosolic Ca^2+^ influx and attenuates cold tolerance in apple

**DOI:** 10.1111/tpj.71182

**Published:** 2026-10-23

**Authors:** Jinzhu Wang, Mengyao Yang, Yehua Li, Lepu Jiang, Yanghao Long, Zhijun Zhang, Lina Qiu, Jie Yang, Fengwang Ma, Ke Mao

**Affiliations:** ^1^ State Key Laboratory for Crop Stress Resistance and High‐Efficiency Production/Shaanxi Key Laboratory of Apple, College of Horticulture Northwest A&F University Yangling Shaanxi China

**Keywords:** apple, cold tolerance, calcium signaling, MdANN2, phosphorylation, MdMPK6a/b

## Abstract

Annexins (ANNs) are Ca^2+^‐dependent phospholipid‐binding proteins that play important roles in plant responses to environmental stress. However, the apple *ANN* gene family and the regulatory mechanisms by which ANNs participate in cold‐stress responses remain poorly understood. In this study, 15 MdANN family members were identified in the apple genome and divided into three subfamilies. Among them, *MdANN2* expression was strongly induced by low temperature. Functional analyses showed that *MdANN2* overexpression markedly enhanced cold tolerance in apple, whereas virus‐induced gene silencing (VIGS) of *MdANN2* compromised cold tolerance. Subcellular localization assays revealed that MdANN2 localized to the plasma membrane, cytoplasm, and nucleus. Non‐invasive micro‐test technology (NMT) further showed that MdANN2 is associated with Ca^2+^‐permeable channel activity: cold stimulation significantly enhanced cytosolic Ca^2+^ influx in *MdANN2*‐overexpressing plants, whereas exogenous application of the Ca^2+^ channel blocker LaCl_3_ or the Ca^2+^ chelator EGTA substantially inhibited this influx. The kinases MdMPK6a and MdMPK6b physically interacted with and phosphorylated MdANN2 both *in vitro* and *in vivo*. Silencing *MdMPK6a/b* significantly increased apple cold tolerance, whereas overexpression of *MdMPK6a/b* reduced apple cold tolerance. Moreover, transient overexpression of *MdMPK6a/b* in the *MdANN2*‐overexpression background attenuated MdANN2‐mediated cold tolerance and Ca^2+^ influx. In addition, co‐localization assays showed that MdMPK6a/b decreased the accumulation of MdANN2 at the plasma membrane. Together, these findings demonstrate that MdANN2 positively regulates apple cold tolerance by promoting cold‐induced Ca^2+^ influx, a process inhibited by MdMPK6a/b‐mediated phosphorylation. This study identifies a novel MdMPK6a/b‐MdANN2 module that governs Ca^2+^ signaling in apple under cold stress, thereby providing promising molecular targets for improving cold tolerance in apple and other crop species.

## Significance Statement

Cold stress severely restricts apple yield and quality, yet the mechanisms linking early calcium signaling with cold‐response regulation remain poorly understood. This study identifies an MdMPK6a/b–MdANN2 module in which ANN2 promotes cold‐induced calcium influx and tolerance, while MPK6a/b‐mediated phosphorylation fine‐tunes this process, providing new insights into calcium signaling regulation in apple and other perennial woody plants.

## INTRODUCTION

Global climate change has increased the unpredictability and severity of temperature extremes. Although mean temperatures are rising, cold waves during critical developmental stages continue to pose serious threats to agricultural production (Ding et al., 2019; Rihan et al., 2017). Low‐temperature stress disrupts multiple physiological and biochemical processes in plants, including membrane fluidity, enzyme activity, reactive oxygen species (ROS) homeostasis, and photosynthetic organ integrity (Kidokoro et al., 2022). These cellular disturbances ultimately lead to growth inhibition, delayed flowering, reduced fruit set, and substantial yield losses (Penfield et al., 2021). As sessile organisms, plants have evolved sophisticated mechanisms to perceive and respond to low temperature (Zhu, 2016). A decrease in ambient temperature is initially perceived through changes in membrane fluidity and transient increases in cytosolic Ca^2+^ concentration. The transition of the plasma membrane from a liquid‐crystalline to a gel‐like phase activates Ca^2+^‐permeable channels, including cyclic nucleotide‐gated channels (CNGCs), glutamate receptor‐like channels (GLRs), hyperosmolality‐gated calcium‐permeable channels (OSCAs), mechanosensitive Ca^2+^ channels (MCAs), and annexins (ANNs) (Demidchik et al., 2018; Lee & Seo, 2021; Park & Shin, 2022). The resulting rapid increase in cytosolic Ca^2+^ generates characteristic calcium signatures that are decoded and relayed through phosphorylation cascades to downstream transcriptional regulators, including the positive regulator ICE1 and the negative regulator MYB15, and ultimately to their target genes, *DREB1/CBFs*, and downstream cold‐responsive (*COR*) genes (Gilmour et al., 1998; Jaglo‐Ottosen et al., 1998; Knight et al., 1996; Liu et al., 1998).

Annexins are Ca^2+^ channels that constitute an evolutionarily conserved protein family found in animals and plants. They bind membrane phospholipids in a Ca^2+^‐dependent manner and contribute to intracellular and extracellular Ca^2+^ homeostasis (Laohavisit & Davies, 2011). Structurally, ANN proteins contain a conserved core domain consisting of four repeats (I–IV), each approximately 70 amino acids in length, together with a variable N‐terminal region that differs in sequence and length among family members (Gerke & Moss, 2002). ANN family size varies among species: eight members in *Arabidopsis thaliana* (Cantero et al., 2006), 10 in rice (*Oryza sativa*) (Jami et al., 2012), nine in crape myrtle (*Lagerstroemia indica*) (Wei et al., 2024), and 12 in poplar (*Populus trichocarpa*) (Wei et al., 2022). A growing body of evidence indicates that ANNs regulate cytosolic Ca^2+^ concentrations and participate in physiological responses to abiotic stress (Clark et al., 2012; Laohavisit et al., 2009; Mortimer et al., 2008). For example, AtANN1 mediates ROS and salt stress‐induced increases in cytosolic Ca^2+^ (Laohavisit et al., 2012; Lee et al., 2004; Richards et al., 2014). AtANN1 and AtANN4 act cooperatively during drought and salt stress, and their functions are influenced by photoperiod (Huh et al., 2010). Overexpression of *OsANN3* improves drought tolerance in rice (Li et al., 2019), and heterologous expression of maize *ZmANN33* and *ZmANN35* enhances cold tolerance in *Arabidopsis* (He et al., 2019). However, the *ANN* gene family and its roles in mediating abiotic stress responses in apple remain uncharacterized.

Post‐translational modifications, especially phosphorylation, profoundly modulate the activity, stability, and subcellular localization of ion transporters, thereby fine‐tuning plant growth, development, and environmental stress responses (Niño‐González & Duque, 2025). For example, the SnRK2‐family kinase OST1/SnRK2.6 and the CDPK‐family kinases CDPK21/23 act synergistically to phosphorylate slow‐type anion channel 1 (SLAC1) at distinct sites, thereby ensuring robust stomatal closure under drought stress (Brandt et al., 2015; Geiger et al., 2009). The CIPK family kinase SOS2/CIPK24 interacts with and phosphorylates the plasma membrane‐localized Na^+^/H^+^ antiporter SOS1, thereby promoting Na^+^ efflux and enhancing plant salt tolerance. Furthermore, this process relies on a salt stress‐triggered Ca^2+^ signal and on Ca^2+^ binding to the Ca^2+^ sensor SOS3/CBL4 (Halfter et al., 2000; Qiu et al., 2002; Quintero et al., 2011). Plant ANNs are also regulated post‐translationally. In *Arabidopsis*, ANN4 mediates salt stress‐triggered Ca^2+^ transients. In response to elevated cytosolic Ca^2+^ levels, the SOS2 kinase interacts with and phosphorylates ANN4 to repress its transporter activity, thereby fine‐tuning the salt‐specific Ca^2+^ signal (Ma et al., 2019). In cotton, GhANN8b undergoes phosphorylation under salt stress and positively modulates salt tolerance by promoting Ca^2+^ influx. Conversely, the phosphatase GhDsPTP3a interacts with and dephosphorylates GhANN8b, thereby negatively regulating GhANN8b‐mediated salt tolerance (Mu et al., 2019). Recent studies have demonstrated that SnRK2‐mediated phosphorylation modulates ANN‐dependent cold stress responses in *Arabidopsis*. The OST1 kinase interacts with and phosphorylates ANN1, thereby enhancing its Ca^2+^ transport activity and reinforcing cold‐induced Ca^2+^ signaling. This cascade further elevates the expression of *CBF* and *COR* genes, ultimately improving plant cold tolerance (Liu et al., 2021). However, whether other kinases participate in ANN‐regulated cold stress responses remains unclear.

Mitogen‐activated protein kinase (MAPK) cascades are central components of plant cold signaling (Colcombet & Hirt, 2008; Guo et al., 2018; Wen et al., 2023). A canonical MAPK cascade comprises three classes of protein kinases: MAPK kinase kinases (MAPKKKs, MAP3Ks, or MEKKs), MAPK kinases (MAP2Ks, MKKs, or MEKs), and MAPKs/MPKs (De Zelicourt et al., 2016). As terminal kinases in the cascade, MPKs directly phosphorylate diverse downstream transcription factors to fine‐tune plant stress responses. In *Arabidopsis*, the MKK4/5‐MPK3/6 cascade negatively regulates cold tolerance by phosphorylating ICE1, which accelerates ICE1 degradation and represses its transcriptional activity (Li et al., 2017; Zhao et al., 2017); a similar mechanism has also been proposed in apple (Yang et al., 2023). Conversely, the MEKK1‐MKK2‐MPK4 cascade positively regulates cold tolerance through the ICE1‐CBF pathway by suppressing MPK3/6 protein abundance and kinase activity (Teige et al., 2004). In tomato, SlMPK1 and SlMPK2 phosphorylate SlBBX17, strengthening its interaction with SlHY5 and thereby enhancing cold tolerance via the CBF‐dependent pathway (Song et al., 2023). MPKs also participate in plant stress responses by phosphorylating diverse functional proteins. For example, *Arabidopsis* MPK3/6 activate stomatal immunity by phosphorylating villin3 (VLN3) to modulate microfilament cytoskeleton remodeling (Zou et al., 2021). In apple, MxMPK4‐1 positively modulates the activity of NADPH oxidase MxRBOHD2 and thereby enhances Fe‐deficiency responses via direct phosphorylation of MxRBOHD2 (Zhai et al., 2022). However, it remains unclear whether MPKs directly phosphorylate Ca^2+^ channels to modulate plant cold‐stress responses.

Apple (*Malus domestica*) is an economically important fruit crop widely cultivated in temperate regions (Verma et al., 2024). However, cold climates restrict its cultivation, and late‐spring frosts can damage floral buds, impair fruit quality, and cause major economic losses (Da Rosa, 2025; Eccel et al., 2009; Guo et al., 2019). Although cold stress is known to trigger transient increases in Ca^2+^ concentrations in the cytosol and organelles, the mechanisms underlying the generation and modulation of cold‐induced Ca^2+^ signals remain largely unknown, particularly those involving ANN proteins in apple. In this study, we identified 15 *ANN* family genes from the apple genome and demonstrated that *MdANN2* is a cold‐responsive gene that positively regulates cold tolerance by promoting cytosolic Ca^2+^ influx. Furthermore, the kinases MdMPK6a and MdMPK6b were found to interact with and phosphorylate MdANN2, inhibiting MdANN2‐mediated Ca^2+^ influx, reducing its plasma‐membrane accumulation, and thereby attenuating cold tolerance in apple. These findings enrich the regulatory network underlying plant responses to cold stress and provide a molecular basis for improving cold tolerance in apple via genetic modification.

## RESULTS

### Genome‐wide identification, bioinformatic analysis, and cold‐induced expression profiling of *MdANN* genes

Based on HMMER screening and BLAST searches using *Arabidopsis* annexin (AtANN) proteins as queries (Table S1), a total of 15 *MdANN* genes were identified in the apple genome. These genes were named *MdANN1*‐*MdANN15* according to their chromosomal positions (Table S2). Physicochemical properties of the 15 MdANN proteins, including protein length, predicted molecular weight, pI, and instability index, were also analyzed. The 15 MdANN proteins ranged from 284 to 359 amino acids in length, with predicted molecular weights of 33.05–40.60 kDa and theoretical isoelectric points (pI) ranging from 6.03 to 9.18 (Table S2). Their instability indices ranged from 26.38 to 47.46, with 10 of the 15 MdANN proteins exhibiting values below 40, suggesting that most members are predicted to be relatively stable (Table S2). Chromosomal localization analysis revealed that the 15 *MdANN* genes were distributed across nine of the 17 apple chromosomes, and three pairs of tandem‐duplicated genes were identified (Figure S1A; Figure 1A). Additionally, collinearity analysis identified three pairs of segmentally duplicated genes (Figure 1A), indicating that both tandem and segmental duplication events contributed substantially to the expansion of the ANN family in apple.

**Figure 1:**
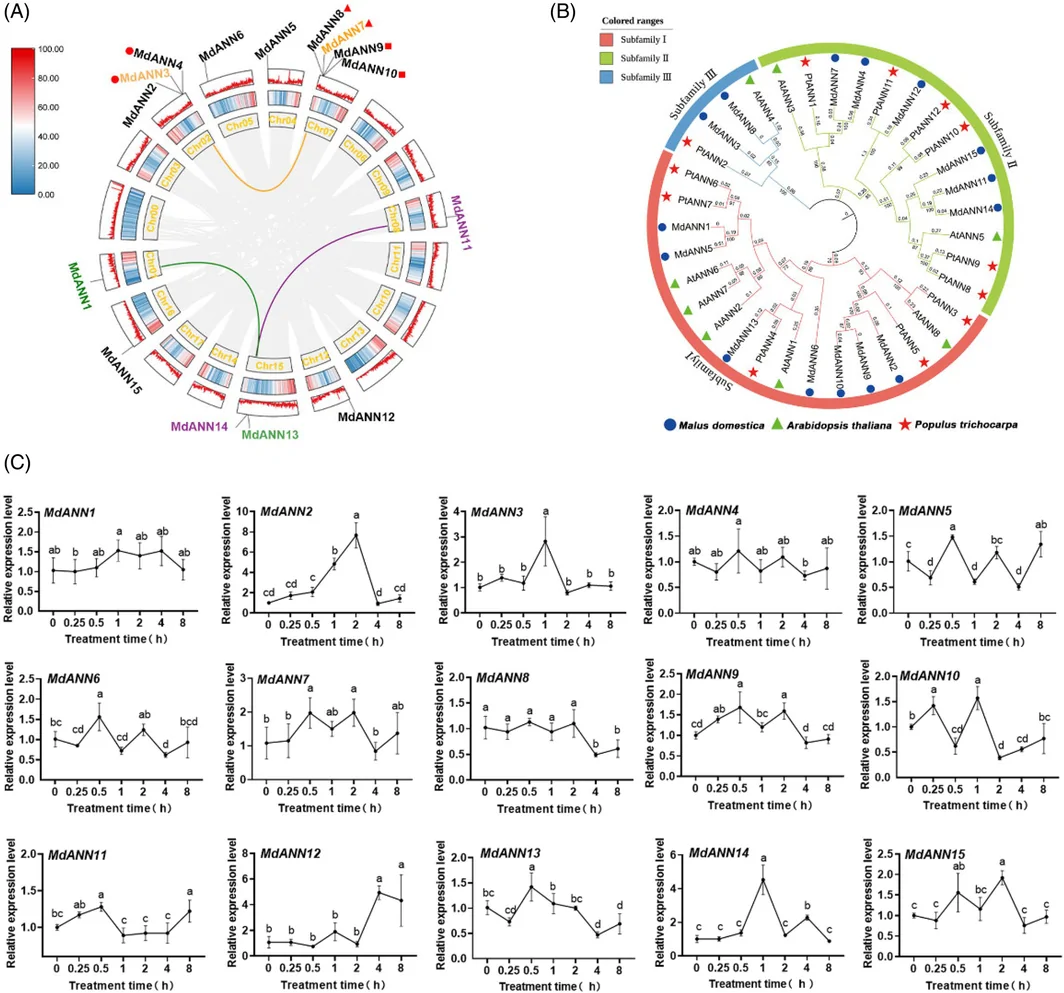
Genome‐wide distribution, phylogenetic relationships, and cold‐induced expression of apple *ANN* genes. (A) Chromosomal distribution and collinearity of the 15 *ANN* family genes in apple. Colored lines and graphical annotations indicate segmentally or tandemly duplicated gene pairs, and gray lines denote collinear blocks. Chr01 to Chr17 represent the 17 apple chromosomes, and Chr00 represents unanchored genomic scaffolds. (B) Phylogenetic analysis of ANN proteins from apple, poplar, and Arabidopsis. The phylogenetic tree was constructed in MEGA v7.0 using the maximum‐likelihood method. Roman numerals indicate the three ANN subfamilies. At, *Arabidopsis thaliana*; Md, *Malus domestica*; Pt, *Populus trichocarpa*. Numbers at the nodes indicate bootstrap support values. Gene IDs are listed in Table S1. (C) Relative transcript levels of *MdANN* genes in apple leaves after cold treatment (4°C for 8 h). Values are means ± SD from three biological replicates. Different letters indicate significant differences as determined by one‐way ANOVA followed by Duncan's test (*P* < 0.05).

To examine the phylogenetic relationships among apple ANN proteins, a phylogenetic tree was constructed using the 15 MdANNs, eight *Arabidopsis* AtANNs, and 12 poplar PtANNs (Table S1). The analysis grouped MdANN proteins into three subfamilies: I, II, and III, among which subfamily I contained the largest number of MdANN members (Figure 1B). To characterize the structural features of MdANNs, their gene structures and conserved motifs were analyzed (Figure S1B). The exon‐intron and conserved motif compositions of *MdANNs* within the same subfamily exhibited similar patterns, while marked differences were observed among subfamilies. In particular, subfamily III lacked motifs 1 and 8 and possessed an additional motif 10. *MdANN5* and *MdANN12* diverged markedly from other members because they lacked core motifs such as motif 3, suggesting that the genome‐predicted coding sequences of these two genes might be inaccurate. Three‐dimensional structure prediction was performed to further characterize MdANN protein structural features (Figure S1C). The predicted MdANN structures were highly similar to those of previously characterized Ca^2+^‐transporting ANN proteins in other plants (Wei et al., 2022, 2024), indicating their potential functions as Ca^2+^ transporters. MdANN members within the same subfamily exhibited more similar structures than those from different subfamilies, further supporting the phylogenetic classification (Figure S1B; Figure 1C).

To investigate the roles of apple *ANN* genes in cold stress responses, the expression patterns of the 15 *MdANNs* in apple plants during cold treatment were analyzed. Several *MdANN* genes, including *MdANN2*, *MdANN3*, and *MdANN14*, were rapidly induced by cold stress (Figure 1C). Among them, *MdANN2* showed the strongest transcriptional response, with its expression increasing by more than sevenfold at 2 h, substantially exceeding the maximal induction observed for other *MdANNs*. Based on its particularly strong cold responsiveness, *MdANN2* was therefore selected as the representative for subsequent functional analyses. Subcellular localization assays showed that MdANN2 was localized to the plasma membrane (Figure S2), implying its potential role in Ca^2+^ transmembrane transport. MdANN2‐GFP signals were also detected in the cytoplasm and nucleus, indicating that its Ca^2+^ transport capacity may be regulated by subcellular distribution.

### *MdANN2* positively regulates cold tolerance in apple

To clarify the function of *MdANN2* in cold tolerance, an *MdANN2* overexpression (OE) vector was constructed and introduced into apple plants. Following screening by genomic PCR and RT‐qPCR, three independent transgenic apple lines (OE#1‐OE#3) overexpressing *MdANN2* were obtained (Figure S3). Freezing treatment showed that, compared with wild‐type (WT) plants, OE lines exhibited less severe leaf injury and wilting and displayed stronger cold tolerance (Figure 2A). Survival rates were significantly higher in OE lines than in WT plants, whereas relative electrolyte leakage in leaves was significantly lower (Figure 2B,C). ROS accumulation and antioxidant enzyme activities were also examined. DAB and NBT staining showed no obvious accumulation of H_2_O_2_ or $O_{2}^{-}$ in leaves under control conditions. After freezing treatment, leaves showed varying degrees of yellow‐brown DAB staining and blue NBT staining, but staining intensity was substantially lower in OE lines than in WT plants. Quantitative measurements of H_2_O_2_ and $O_{2}^{-}$ were consistent with the staining results (Figure 2D–G). Under normal conditions, SOD, POD, and CAT activities did not differ significantly between WT and OE lines. After freezing treatment, the activities of all three antioxidant enzymes increased, and the increases were significantly greater in OE lines than in WT plants (Figure 2H–J). These results indicate that *MdANN2* overexpression enhances cold tolerance in apple plants.

**Figure 2:**
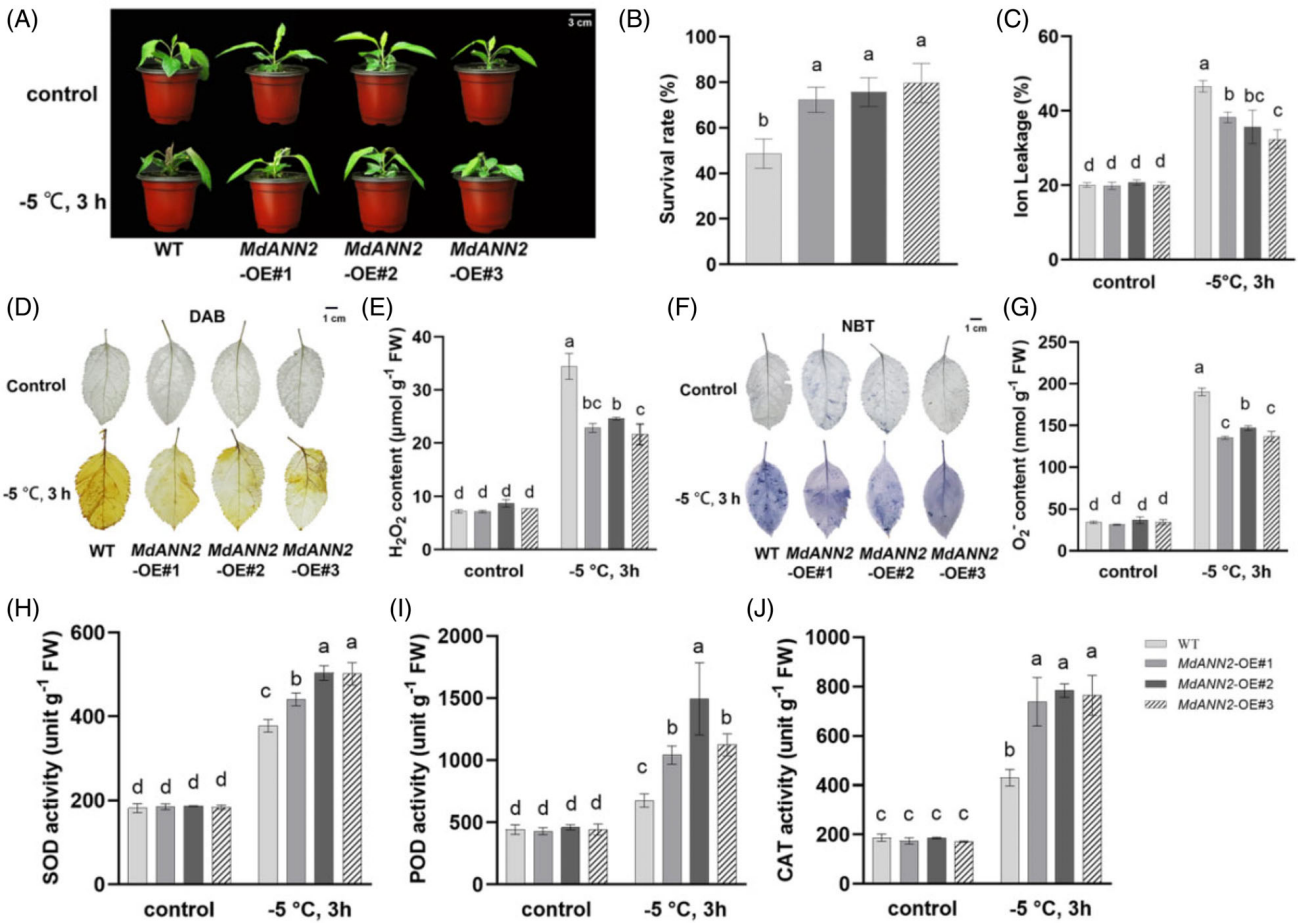
Overexpression of *MdANN2* enhanced freezing tolerance in apple plants. (A) Phenotypes of wild‐type (WT) and *MdANN2*‐overexpressing apple plants before and after freezing treatment. (B) Survival rate. (C) Relative electrolyte leakage. (D) 3,3′‐diaminobenzidine (DAB) staining of leaves. (E) H_2_O_2_ content in apple leaves. (F) Nitro blue tetrazolium (NBT) staining of leaves. (G) $O_{2}^{-}$ content in apple leaves. (H) Superoxide dismutase (SOD) activity. (I) Peroxidase (POD) activity. (J) Catalase (CAT) activity. Values are means ± SD from three biological replicates. Different letters indicate significant differences as determined by one‐way ANOVA followed by Duncan's test (*P* < 0.05).

To further evaluate the role of *MdANN2* in apple cold tolerance, VIGS was used to suppress *MdANN2* expression in apple plants. The transcript abundance of *MdANN2* was significantly lower in leaves of TRV‐*MdANN2* plants compared with TRV control plants (Figure S4). After freezing treatment, TRV‐*MdANN2* plants exhibited more severe leaf damage and wilting relative to control plants, accompanied by markedly lower survival rates and higher relative electrolyte leakage (Figure 3A–C). Furthermore, TRV‐*MdANN2* plants accumulated higher levels of ROS after freezing treatment, whereas their antioxidant enzyme activities were significantly lower than those of TRV controls (Figure 3D–J). These results demonstrate that interfering with *MdANN2* expression reduces cold tolerance in apple plants.

**Figure 3:**
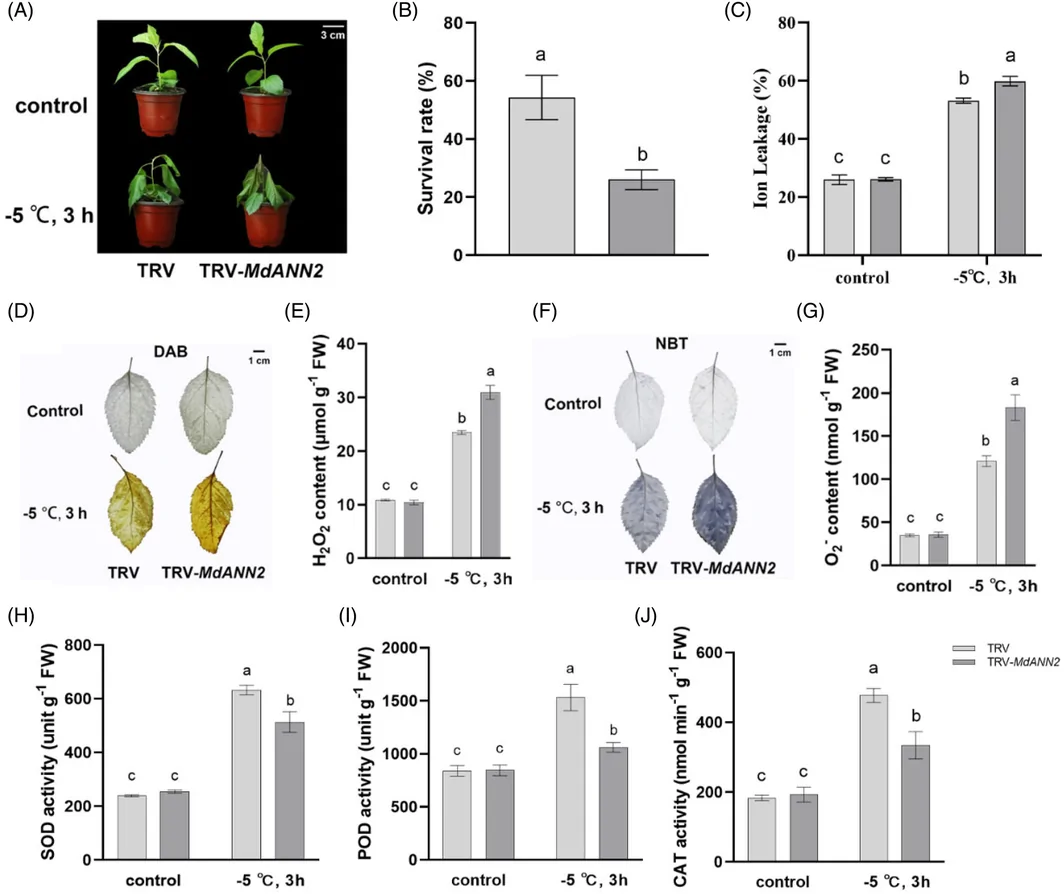
Silencing of *MdANN2* reduced freezing tolerance in apple plants. (A) The phenotypic characteristics of apple plants under freezing treatment. (B) Survival rate. (C) Relative electrolyte leakage. (D) 3,3′‐diaminobenzidine (DAB) staining of leaves. (E) H_2_O_2_ content in apple leaves. (F) Nitro blue tetrazolium (NBT) staining of leaves. (G) $O_{2}^{-}$ content in apple leaves. (H) Superoxide dismutase (SOD) activity. (I) Peroxidase (POD) activity. (J) Catalase (CAT) activity. Values are means ± SD from three biological replicates. Different letters indicate significant differences as determined by one‐way ANOVA followed by Duncan's test (*P* < 0.05).

### MdANN2 promotes cold‐induced Ca^2+^ influx and the expression of cold‐responsive genes

ANNs are generally considered Ca^2+^‐permeable plasma‐membrane components that mediate cytosolic Ca^2+^ influx during plant growth and stress responses, thereby influencing Ca^2+^ signal transduction (Davies, 2014; Wu et al., 2022). To test whether MdANN2 affects cytosolic Ca^2+^ influx in response to cold stress, NMT was used to measure Ca^2+^ fluxes in leaves of WT and *MdANN2*‐OE plants. Cold stimulation rapidly induced cytosolic Ca^2+^ influx, and the influx rate was significantly higher in *MdANN2*‐OE lines than in WT plants. The peak Ca^2+^ influx under cold stress was also significantly higher in OE lines than in WT plants (Figure 4A,B). Furthermore, the MdANN2‐promoted Ca^2+^ influx was substantially suppressed by the Ca^2+^ channel blocker LaCl_3_ or the Ca^2+^ chelator EGTA (Figure 4A,B). These results indicate that MdANN2 promotes cytosolic Ca^2+^ influx in response to cold.

**Figure 4:**
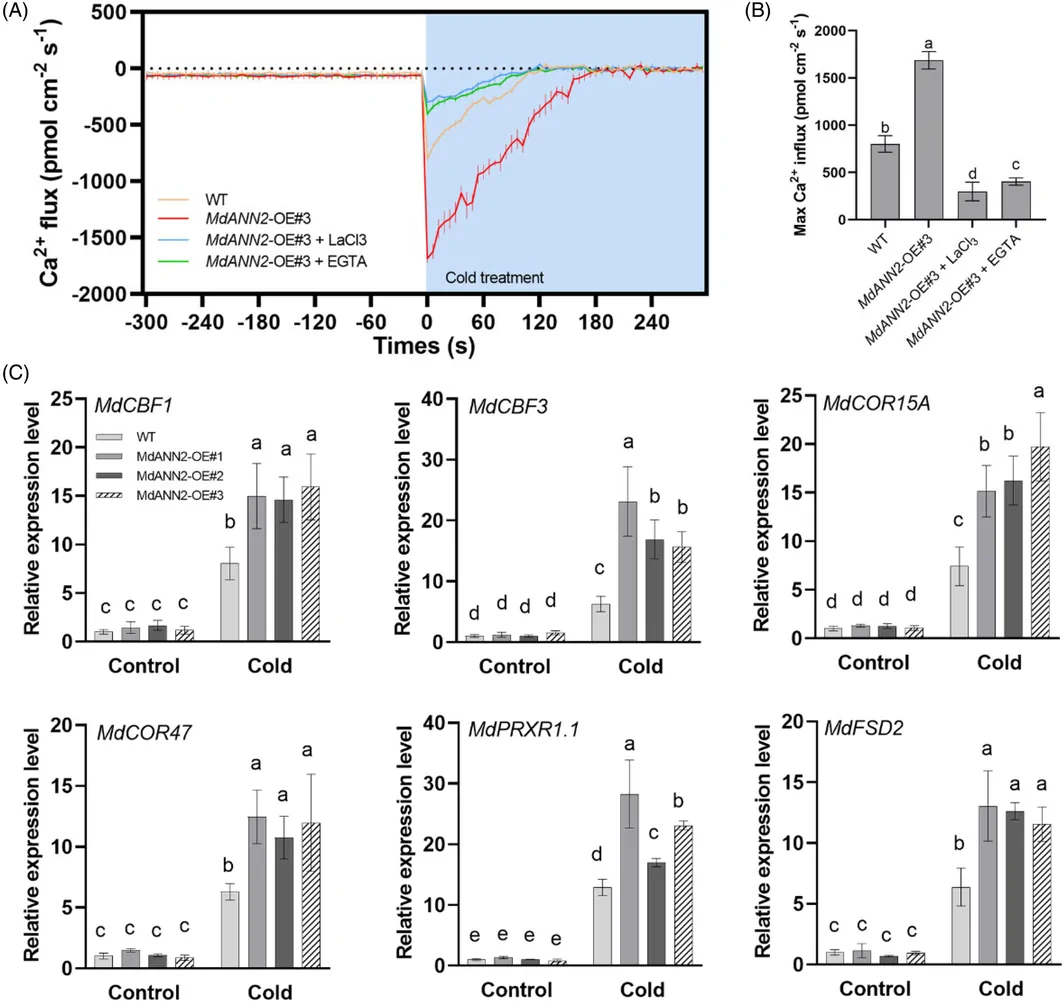
MdANN2 promotes cold‐induced Ca^2+^ influx and the expression of cold‐responsive genes. (A) Net cytosolic Ca^2+^ influx rates in apple leaves under cold stress. Based on the high expression level of *MdANN2* in OE#3, this line was selected for the NMT assay. (B) Maximum Ca^2+^ influx rates. Values are means ± SD from six biological replicates. (C) Relative transcript levels of cold‐responsive genes in apple leaves after cold treatment (4°C for 8 h). Values are means ± SD from three biological replicates. Different letters indicate significant differences as determined by one‐way ANOVA followed by Duncan's test (*P* < 0.05).

The *CBF–COR* pathway serves as a core regulatory mechanism underlying plant cold responses and is activated by cold‐induced Ca^2+^ signaling (Jaglo‐Ottosen et al., 1998; Knight et al., 1996; Liu et al., 2021). Previous studies have shown that several apple cold‐responsive genes are strongly induced by cold stress and participate in ROS scavenging, including *MdCBF1/3*, *MdCOR15A*, *MdCOR47*, and antioxidant enzyme‐encoding genes *MdFSD2* and *MdPRXR1.1* (Mei et al., 2023). Therefore, the expression levels of these genes were examined. Under normal conditions, their transcript levels remained at basal levels and did not differ significantly among genotypes. Cold treatment significantly induced the expression of these genes; however, their expression was significantly higher in *MdANN2*‐OE lines compared with WT plants (Figure 4C). These results indicate that *MdANN2* positively regulates the response to cold stress in apple.

### MdMPK6a and MdMPK6b interact with and phosphorylate MdANN2

To dissect the molecular mechanism underlying the role of MdANN2 in the apple cold response, we performed yeast two‐hybrid (Y2H) screening using MdANN2 as bait to identify its interacting partners. This screen identified a protein kinase, MdMPK6a (MD15G1147300) (Table S4), which our previous study suggested may act as a negative regulator of the apple cold response (Yang et al., 2023). Because MdMPK6a has another highly homologous paralog, MdMPK6b (Yang et al., 2023), we further examined the interactions between MdANN2 and MdMPK6a/b using Y2H assays. Yeast cells co‐transformed with *MdANN2*‐pPR3‐N and *MdMPK6a*‐pDHB1 (or *MdMPK6b*‐pDHB1) vectors survived on SD/−Trp/−Leu/‐His/−Ade medium and turned blue upon supplementation with X‐α‐Gal, whereas none of the control combinations showed visible growth (Figure 5A), demonstrating direct interactions between MdMPK6a/b and MdANN2 in yeast cells. Consistently, split‐luciferase complementation assays detected strong luminescence signals in *Nicotiana benthamiana* leaves co‐expressing *MdMPK6a/b*‐nLUC and *MdANN2*‐cLUC, whereas no luminescence was observed in control groups (Figure 5B,C). Furthermore, bimolecular fluorescence complementation (BiFC) assays detected intense yellow fluorescence signals at the plasma membrane and in the cytoplasm of *N. benthamiana* leaf cells co‐expressing *MdMPK6a/b*‐nYFP and *MdANN2*‐cYFP, while no fluorescence was observed in any control combination (Figure 5D,E). To further validate the MdANN2‐MdMPK6a/b interaction *in vitro*, we performed pull‐down assays. The results showed that both recombinant MdMPK6a‐GST and MdMPK6b‐GST, but not GST alone, were pulled down by MdANN2‐His (Figure 5F,G). Together, these results demonstrate that MdANN2 interacts with MdMPK6a/b both *in vivo* and *in vitro*.

**Figure 5:**
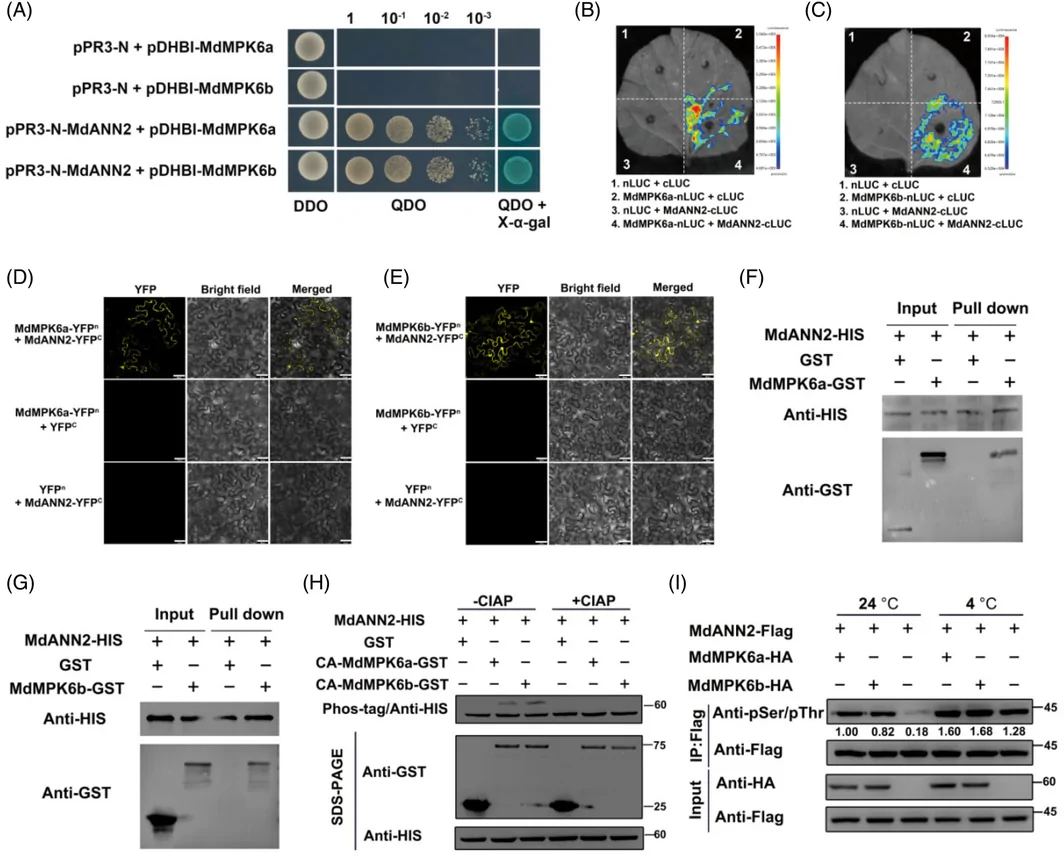
MdMPK6a/b physically interact with and phosphorylate MdANN2. (A) Yeast two‐hybrid (Y2H) assays showing the interaction between MdMPK6a/b and MdANN2 in yeast cells. Yeast cells co‐transformed with pPR3‐N‐MdANN2 and pDHBI‐MdMPK6a/b were grown on SD/−Leu/−Trp (DDO) or SD/−Leu/−Trp/−His/−Ade (QDO) medium. (B, C) Split‐luciferase complementation assays showing the interaction between MdMPK6a/b and MdANN2 in *Nicotiana benthamiana* leaves. (D, E) Bimolecular fluorescence complementation (BiFC) assays showing the interaction between MdMPK6a/b and MdANN2. *MdMPK6a/b*‐nYFP and *MdANN2*‐cYFP were co‐expressed in *N. benthamiana* leaves, and YFP fluorescence was detected by confocal microscopy. Scale bar = 50 μm. (F, G) Pull‐down assays confirming the interaction between MdMPK6a/b and MdANN2 *in vitro*. (H) Phos‐tag assays showing that MdMPK6a/b phosphorylates MdANN2 *in vitro*. Calf intestinal alkaline phosphatase (CIAP) was used to remove protein phosphorylation. (I) *In vivo* phosphorylation assays showing that MdANN2 is phosphorylated by MdMPK6a/b. *MdMPK6a/b*‐HA and *MdANN2*‐Flag were co‐expressed in apple calli for 3 days, followed by treatment at 24 or 4°C for 8 h. After total protein extraction and immunoprecipitation, phosphorylated MdANN2 was detected using an anti‐pSer/pThr antibody. Grayscale intensities of protein bands were quantified using ImageJ. IP: Flag was used as the loading control, and the relative protein band intensity in the first lane was normalized to 1. All experiments were independently repeated three times with similar results, and a representative image is shown.

Given the physical interactions between MdMPK6a/b and MdANN2, we further investigated whether MdMPK6a/b could phosphorylate MdANN2. *In vitro* kinase assays were performed using purified MdANN2‐His and constitutively active CA‐MdMPK6a/b‐GST recombinant proteins. The results showed that MdMPK6a and MdMPK6b each directly phosphorylated MdANN2. Moreover, the phosphorylation signal disappeared after treatment with calf intestinal alkaline phosphatase (CIAP), confirming the specificity of this phosphorylation event (Figure 5H). Furthermore, *in vivo* phosphorylation assays were performed by co‐expressing MdMPK6a/b‐HA and MdANN2‐Flag in apple ‘Orin’ calli. Consistent with the *in vitro* results, MdMPK6a and MdMPK6b were capable of phosphorylating MdANN2 *in vivo*, and the phosphorylation level of MdANN2 was enhanced under cold conditions (Figure 5I). Taken together, our results demonstrate that MdMPK6a/b physically interact with and phosphorylate MdANN2.

### MdMPK6a/b negatively regulate apple cold tolerance

To explore the biological functions of *MdMPK6a/b* in apple cold responses, VIGS was used to suppress their expression (Figure S5). Under normal growth conditions, no obvious phenotypic differences were observed between *MdMPK6a/b*‐silenced plants and TRV control plants. After freezing treatment, *MdMPK6a/b*‐silenced plants exhibited less severe leaf injury and wilting than control plants, indicating enhanced cold tolerance (Figure 6A). Consistently, *MdMPK6a/b*‐silenced plants exhibited a significantly higher survival rate and lower leaf relative electrolyte leakage than control plants (Figure 6B,C). ROS accumulation and antioxidant enzyme activities in leaves were also measured. DAB and NBT staining and quantitative assays showed that *MdMPK6a/b*‐silenced plants had significantly lower levels of H_2_O_2_ and $O_{2}^{-}$ and higher SOD, POD, and CAT activities than control plants after freezing treatment (Figure 6D–J). To further determine the roles of *MdMPK6a/b* in apple cold tolerance, *MdMPK6a*‐OE and *MdMPK6b*‐OE plants were generated by transient transformation. RT‐qPCR analysis confirmed significantly elevated *MdMPK6a* and *MdMPK6b* transcript levels in the corresponding overexpression plants (Figure S6A,B). Following treatment at −5°C for 3 h, *MdMPK6a*‐OE and *MdMPK6b*‐OE plants exhibited more severe cold injury than WT plants, as indicated by lower survival rates, increased ion leakage and ROS accumulation, and reduced SOD, POD, and CAT activities (Figure S6C–L). These results demonstrate that MdMPK6a/b play a negative role in apple cold tolerance.

**Figure 6:**
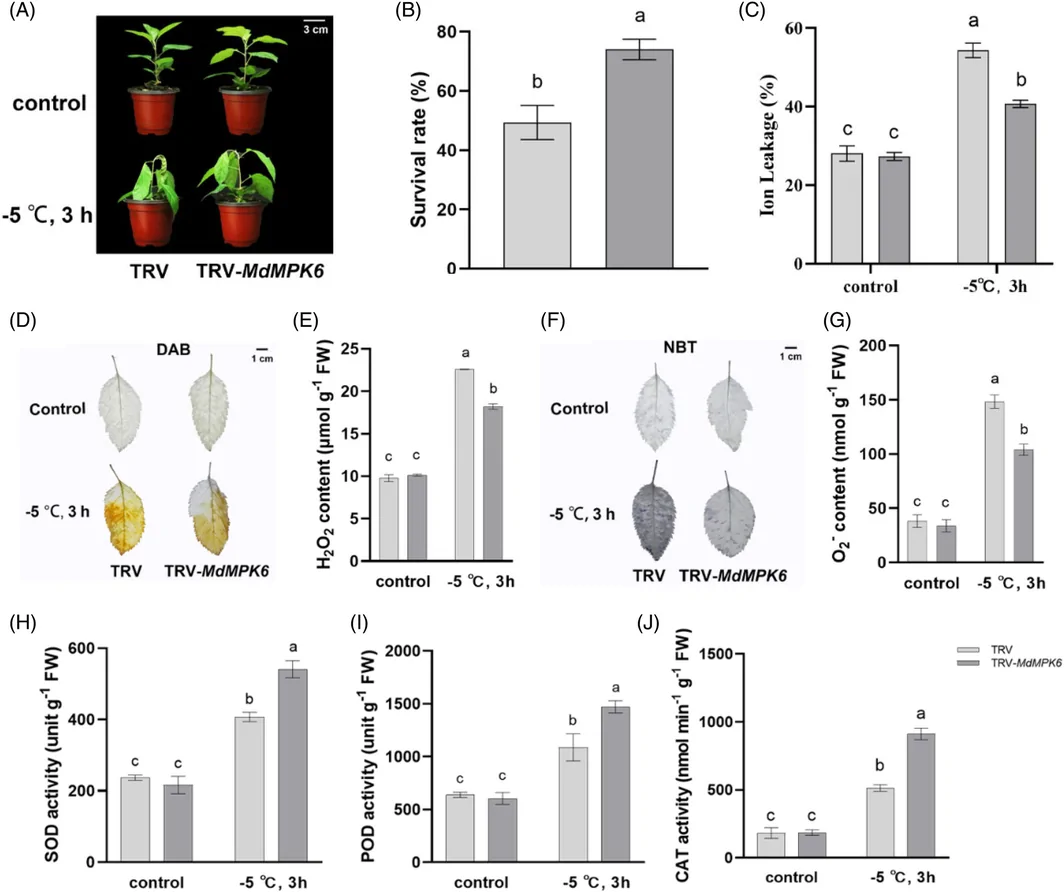
Silencing of *MdMPK6* enhanced freezing tolerance in apple plants. (A) The phenotypic characteristics of apple plants under freezing treatment. (B) Survival rate. (C) Relative electrolyte leakage. (D) 3,3′‐diaminobenzidine (DAB) staining of leaves. (E) H_2_O_2_ content in apple leaves. (F) Nitro blue tetrazolium (NBT) staining of leaves. (G) $O_{2}^{-}$ content in apple leaves. (H) Superoxide dismutase (SOD) activity. (I) Peroxidase (POD) activity. (J) Catalase (CAT) activity. Values are means ± SD from three biological replicates. Different letters indicate significant differences as determined by one‐way ANOVA followed by Duncan's test (*P* < 0.05).

### *MdMPK6a/b*‐overexpression suppresses *MdANN2* ‐mediated cold tolerance

To investigate whether MdMPK6a/b affect MdANN2‐mediated cold tolerance, *MdMPK6a* and *MdMPK6b* were transiently overexpressed separately in *MdANN2*‐OE#3 plants (Figure S7). Phenotypic observations and physiological measurements revealed that the double transgenic plants were more tolerant to freezing stress than the WT. However, overexpression of either *MdMPK6a* or *MdMPK6b* significantly compromised the enhanced freezing tolerance conferred by *MdANN2* overexpression. Compared with *MdANN2*‐OE#3 plants, *MdANN2*‐OE#3 + *MdMPK6a*‐OE and *MdANN2*‐OE#3 + *MdMPK6b*‐OE plants displayed more severe leaf injury and wilting, a markedly lower survival rate, and significantly higher leaf ion leakage after freezing treatment (Figure 7A–C). Consistent with these findings, analyses of ROS accumulation showed that the levels of H_2_O_2_ and $O_{2}^{-}$ in the leaves of *MdANN2*‐OE#3 + *MdMPK6a*‐OE and *MdANN2*‐OE#3 + *MdMPK6b*‐OE double transgenic plants were significantly higher than those in *MdANN2*‐OE#3 plants but remained lower than those in WT plants (Figure 7D–G). In contrast, the activities of SOD, POD, and CAT exhibited the opposite trend in these plants under cold treatment (Figure 7H–J). Taken together, these results demonstrate that both MdMPK6a and MdMPK6b substantially attenuate *MdANN2*‐mediated cold tolerance in apple.

**Figure 7:**
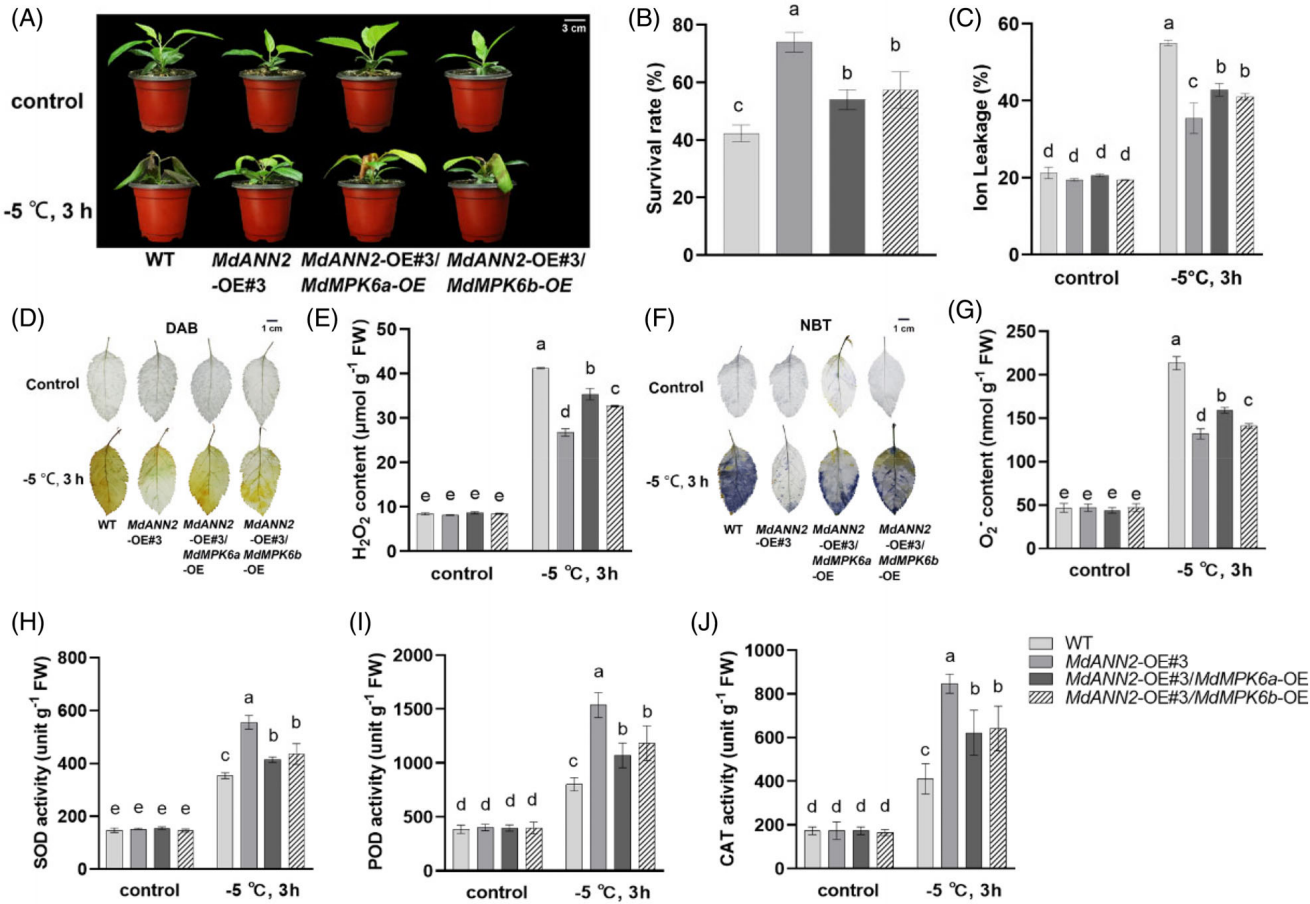
MdMPK6a/b suppresses MdANN2‐mediated freezing tolerance in apple. (A) Phenotypes of apple plants before and after freezing treatment. (B) Survival rate. (C) Relative electrolyte leakage measured before and after freezing treatment. (D) 3,3′‐diaminobenzidine (DAB) staining of leaves. (E) H_2_O_2_ content in apple leaves. (F) Nitro blue tetrazolium (NBT) staining of leaves. (G) $O_{2}^{-}$ content in apple leaves. (H) Superoxide dismutase (SOD) activity. (I) Peroxidase (POD) activity. (J) Catalase (CAT) activity. Values are means ± SD from three biological replicates. Different letters indicate significant differences as determined by one‐way ANOVA followed by Duncan's test (*P* < 0.05).

### MdMPK6a/b inhibit MdANN2‐mediated Ca^2+^ influx and cold response

To examine the regulatory effect of MdMPK6a/b on MdANN2‐associated Ca^2+^ channel activity, Ca^2+^ fluxes in leaves of *MdANN2*‐OE and *MdANN2*‐OE + *MdMPK6a/b*‐OE double‐transgenic apple plants were measured by NMT. After cold stimulation, cytosolic Ca^2+^ influx rates in mesophyll cells were significantly lower in *MdANN2*‐OE + *MdMPK6a*‐OE and *MdANN2*‐OE + *MdMPK6b*‐OE plants than in *MdANN2*‐OE plants (Figure 8A). Peak Ca^2+^ influx was also significantly reduced by *MdMPK6a/b* overexpression in the *MdANN2*‐OE background (Figure 8B). To further explore the influence of *MdMPK6a/b* on the *MdANN2*‐mediated cold response, we determined the expression of *COR* genes in these transgenic apple plants. Under normal conditions, the transcript levels of these genes were comparable among genotypes. Upon cold treatment, *COR* genes were significantly upregulated in *MdANN2*‐OE leaves compared with WT leaves. Notably, this *MdANN2*‐induced upregulation of *COR* genes was significantly repressed in *MdANN2*‐OE + *MdMPK6a*‐OE and *MdANN2*‐OE + *MdMPK6b*‐OE plants (Figure 8C). These data indicate that MdMPK6a and MdMPK6b suppress MdANN2‐mediated Ca^2+^ influx and *COR* gene expression under cold conditions.

**Figure 8:**
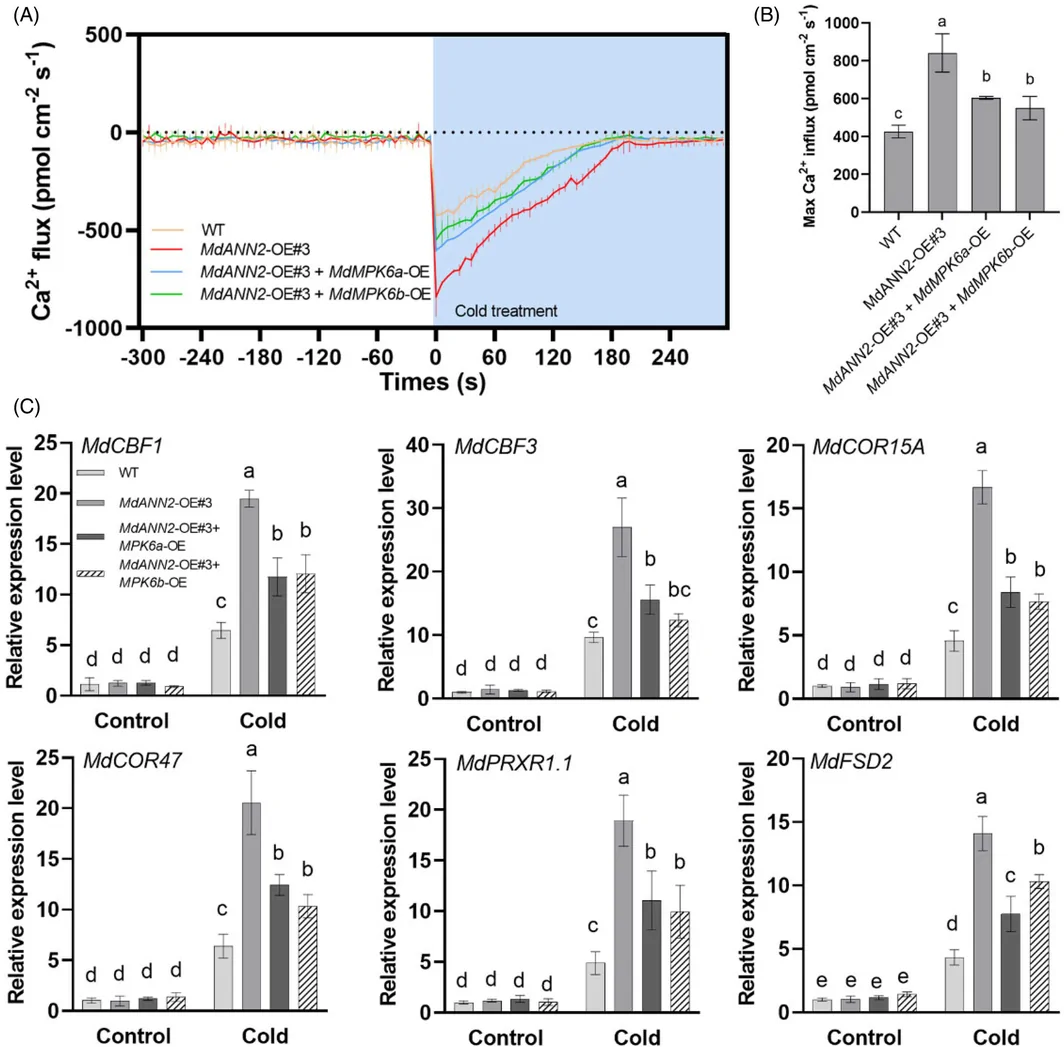
MdMPK6a/b suppress MdANN2‐mediated enhancement of cold‐induced Ca^2+^ influx and cold‐responsive gene expression. (A) Net cytosolic Ca^2+^ influx rates in apple leaves under cold stress. (B) Maximum Ca^2+^ influx rates. Values are means ± SD from six biological replicates. (C) Relative transcript levels of cold‐responsive genes in apple leaves after cold treatment (4°C for 8 h). Values are means ± SD from three biological replicates. Different letters indicate significant differences as determined by one‐way ANOVA followed by Duncan's test (*P* < 0.05).

### MdMPK6a/b reduce the accumulation of MdANN2 at the plasma membrane

Phosphorylation represents an important regulatory mechanism controlling the membrane‐binding capacity of ANN proteins and their membrane‐associated functions (Deora et al., 2004; Konopka‐Postupolska et al., 2011; Laohavisit & Davies, 2011; Zheng et al., 2011). Given that MdMPK6a/b interact with and phosphorylate MdANN2, we hypothesized that MdMPK6a/b may alter the subcellular localization of MdANN2. Subcellular co‐localization assays in *N. benthamiana* leaves revealed that MdANN2 was predominantly localized to the plasma membrane and cytoplasm, with partial nuclear distribution. Notably, when *MdMPK6a*‐HA or *MdMPK6b*‐HA was co‐expressed with *MdANN2*‐GFP, the co‐localization signal of *MdANN2*‐GFP with the plasma‐membrane marker AtCBL1‐mCherry was substantially reduced, indicating decreased plasma membrane localization of MdANN2 (Figure 9A). To further validate this observation, we separated the plasma‐membrane and cytosolic protein fractions of leaf cells. Immunoblot assays showed that co‐expression of either *MdMPK6a*‐HA or *MdMPK6b*‐HA with *MdANN2*‐GFP reduced MdANN2 abundance in the membrane fraction and increased its abundance in the cytosolic fraction (Figure 9B,C). These results collectively demonstrate that MdMPK6a and MdMPK6b inhibit the plasma membrane accumulation of MdANN2.

**Figure 9:**
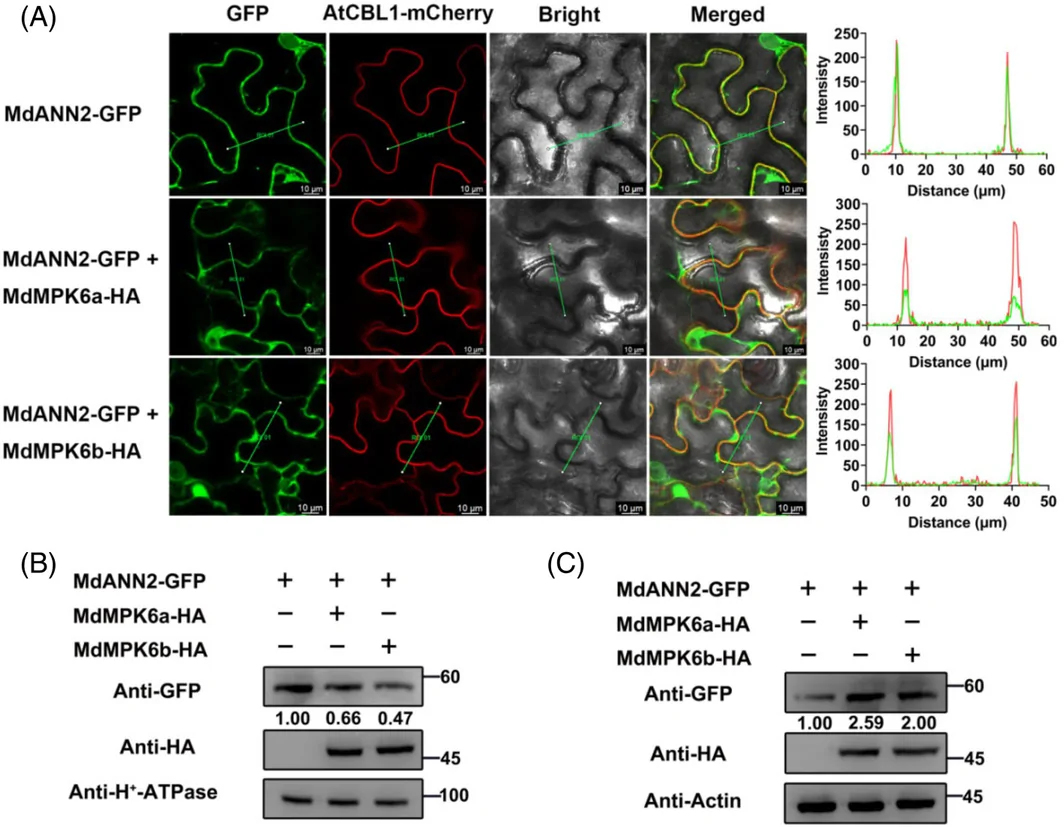
MdMPK6a/B reduce the accumulation of MdANN2 at the plasma membrane. (A) Confocal microscopy showing that MdMPK6a/b decrease the plasma‐membrane localization of MdANN2. Scale bar = 10 μm. AtCBL1‐mCherry, a plasma membrane marker; GFP, green fluorescent protein. Fluorescence intensity was quantified using LAS X software, and the y axis indicates relative pixel intensity. (B) Immunoblot analysis showing that MdMPK6a/b reduce the abundance of MdANN2 in the membrane fraction of *Nicotiana benthamiana* leaves. (C) Immunoblot analysis showing that MdMPK6a/b increase the abundance of MdANN2 in the cytosolic fraction of *N. benthamiana* leaves. Grayscale intensities of protein bands were quantified using ImageJ. H^+^‐ATPase and Actin were used as the loading control, and the relative protein band intensity in the first lane was normalized to 1. All experiments were independently repeated three times with similar results, and a representative image is shown.

## DISCUSSION

ANNs function as Ca^2+^‐permeable channels and participate in plant stress responses. Genome‐wide identification of *ANN* gene families has been extensively reported in several plant species, including *Arabidopsis*, rice, poplar, and crape myrtle (Cantero et al., 2006; Jami et al., 2012; Wei et al., 2022, 2024). However, the *ANN* gene family in apple has not been systematically characterized. In the present study, we identified 15 *MdANN* members from the apple genome for the first time (Table S2). Compared with the previously reported eight *ANN* genes in *Arabidopsis*, nine *LiANN* genes in crape myrtle, ten *OsANN* genes in rice, and twelve *PtANN* genes in poplar, the larger number of *MdANN* members suggests that the apple ANN family may have undergone expansion and functional diversification (Cantero et al., 2006; Jami et al., 2012; Wei et al., 2022, 2024). Collinearity analysis revealed three pairs of segmentally duplicated genes and three tandem duplication events within the MdANN family (Figure 1A), indicating that both segmental and tandem duplications have jointly contributed to the expansion of the ANN family in apple. Phylogenetic, gene structure, and conserved motif analyses further showed that *MdANN* members within the same clade generally shared similar exon–intron organization and motif composition, whereas marked differences were observed among different clades (Figure 1B). These findings suggest that, although MdANN proteins retain the typical conserved ANX domains, divergence in the N‐terminal region, gene structure, and specific functional sites may have contributed to their functional differentiation (Clark et al., 2012; Gerke & Moss, 2002).

Previous studies have demonstrated that ANNs from multiple plant species play important roles in cold‐stress responses (Tong et al., 2025; Xie et al., 2025). Among the 15 *MdANN* genes, *MdANN2* showed the strongest transcriptional induction under cold stress (Figure 1C), suggesting that it may have a particularly important role in the cold‐stress response of apple. Freezing‐stress assays further demonstrated that *MdANN2* positively regulates cold tolerance in apple. Under freezing conditions, *MdANN2*‐overexpressing plants displayed higher survival rates and lower relative electrolyte leakage than wild‐type plants (Figure 2A–C), whereas *MdANN2*‐silenced plants exhibited the opposite phenotypes (Figure 3A–C). These results are consistent with previous studies showing that *AtANN1/4*, *OsANN3/5*, and *ZmANN33/35* positively regulate cold‐stress responses in plants (Liu et al., 2021; Niu et al., 2023; Que et al., 2023; Shen et al., 2017). Plant annexins also participate in abiotic stress responses by regulating antioxidant defense and ROS homeostasis. In rice, *OsANN1* enhances heat tolerance by increasing SOD and CAT activities, thereby maintaining H_2_O_2_ homeostasis and cellular redox balance (Qiao et al., 2015). In *Arabidopsis*, *AtANN1* positively regulates basal and acquired freezing tolerance, and it has been proposed that its contribution to basal freezing tolerance may be associated with the prevention of excessive ROS production (Liu et al., 2021). In agreement with these observations, overexpression of *MdANN2* reduced the accumulation of H_2_O_2_ and $O_{2}^{-}$ and increased antioxidant enzyme activities in apple plants under freezing stress (Figure 2D–J). Conversely, silencing of *MdANN2* resulted in greater ROS accumulation and lower antioxidant enzyme activities (Figure 3D–J). These findings support a model in which *MdANN2* positively regulates apple freezing tolerance by strengthening the antioxidant defense system and maintaining ROS homeostasis.

ANN proteins are Ca^2+^‐dependent phospholipid‐binding proteins that associate with membrane phospholipids and participate in Ca^2+^ homeostasis, thereby playing important roles in plant stress responses (Clark et al., 2012; Laohavisit et al., 2012; Laohavisit & Davies, 2011). In *Arabidopsis*, *AtANN1* mediates ROS‐ and salt stress‐induced increases in cytosolic Ca^2+^ and contributes to abiotic stress responses (Laohavisit et al., 2009; Richards et al., 2014). To determine whether MdANN2 also participates in Ca^2+^ transport in apple, we measured Ca^2+^ fluxes under cold stimulation using NMT. *MdANN2*‐overexpressing plants exhibited a higher Ca^2+^ influx rate and a greater peak influx than WT plants after cold stimulation, suggesting that MdANN2 positively modulates cold‐induced Ca^2+^ influx. This process was markedly inhibited by LaCl_3_ and EGTA (Figure 4A,B), indicating that MdANN2‐mediated cold‐induced Ca^2+^ signaling depends on extracellular Ca^2+^ entry and Ca^2+^ channel activity. Cold‐induced Ca^2+^ signals can activate downstream *CBF* and *COR* gene expression. AtANN1 has been shown to promote cold‐induced Ca^2+^ influx and enhance *CBF* and *COR* expression, thereby improving freezing tolerance (Liu et al., 2021). Similarly, our results showed that *MdANN2* enhanced the expression of apple CBF and *COR* genes under cold stress (Figure 4C). *MdFSD2* and *MdPRXR1.1* are two antioxidant enzyme‐encoding genes that are directly activated by MdNAC104 through a CBF‐independent pathway in response to cold stress (Mei et al., 2023). Although no evidence indicates that these two genes are regulated by Ca^2+^ signaling under cold stress, both genes are positively regulated by *MdANN2* in our study (Figure 4C). This finding suggests that *MdFSD2* and *MdPRXR1.1* may be activated by *MdANN2*‐mediated Ca^2+^ signaling upon cold exposure, although the underlying mechanism requires further investigation. Nevertheless, the coordinated expression of these genes indicates that *MdANN2*‐mediated Ca^2+^ influx is associated not only with the classical CBF‐dependent cold‐response pathway but also with enhanced ROS‐scavenging capacity under cold stress.

Phosphorylation can affect the activity, stability, and subcellular localization of transport proteins. As a major phosphorylation‐based signaling system in plants, MAPK cascades play important roles in cold responses (Colcombet & Hirt, 2008; Guo et al., 2018). Y2H screening identified the MPK family kinases MdMPK6a and MdMPK6b as MdANN2‐interacting proteins capable of directly phosphorylating MdANN2 (Figure 5). Further analyses revealed the negative roles of MdMPK6a/b in MdANN2‐mediated Ca^2+^ influx, *COR* gene expression, and, consequently, cold tolerance in apple (Figures 6, 7, 8; Figure S6). These findings are consistent with the reported role of MPK3/6 in restricting excessive activation of the CBF pathway during cold responses (Li et al., 2017; Zhao et al., 2017). Rapid Ca^2+^ influx is required to initiate defense responses during the early stages of cold stress; however, prolonged or excessive Ca^2+^ signaling may disrupt ion homeostasis, increase metabolic costs, and inhibit growth (Rahmati Ishka et al., 2021; Urquhart et al., 2007). By phosphorylating MdANN2 and reducing its accumulation at the plasma membrane (Figure 9), MdMPK6a/b may modulate the strength of cold‐induced Ca^2+^ signaling, thereby balancing defense activation with signaling homeostasis. Because MPK6‐mediated phosphorylation can also affect substrate stability in some systems (Li et al., 2017; Zhao et al., 2017), whether MdMPK6a/b further regulates MdANN2 protein stability remains to be determined. In addition, the partial nuclear localization of MdANN2 suggests that its function may not be restricted to Ca^2+^ transport at the plasma membrane (Figure S2). Although ANN proteins are generally considered membrane‐binding proteins, some plant ANN members exhibit diverse subcellular localization patterns and may participate in signaling processes beyond the membrane system (Baucher et al., 2011; Lichocka et al., 2018). Indeed, nuclear localization has been reported for several plant annexins. In pea, annexin proteins were detected in the nucleus, nuclear envelope, nucleolus, and chromatin fractions, while the stress‐ and ABA‐responsive alfalfa annexin AnnMs2 was predominantly localized in the nucleolus (Clark et al., 1998; Kovács et al., 1998). Similarly, Arabidopsis ANN5 localizes to the nucleus and nucleolus and has been proposed to participate in the coordination of nuclear and plastid activities (Lichocka et al., 2018), raising the possibility that the partial nuclear localization of MdANN2 may also reflect additional roles in nuclear‐associated cold signaling beyond its function in Ca^2+^ transport. Future studies using nuclear‐localization‐deficient or plasma‐membrane‐targeted *MdANN2* variants may help distinguish its membrane‐associated Ca^2+^ transport function from its potential nuclear regulatory roles.

Integrating published data and our findings, we propose a regulatory module whereby MdMPK6a/b interact with MdANN2 to fine‐tune cold‐induced Ca^2+^ signaling and thereby regulate the cold response in apple (Figure 10). Upon cold exposure, *MdANN2* expression is rapidly induced, and MdANN2 acts as a Ca^2+^‐permeable component at the plasma membrane to promote Ca^2+^ influx, generate cytosolic Ca^2+^ signals, activate CBF‐dependent cold‐responsive transcription, and enhance antioxidant defense. MdMPK6a/b interact with and phosphorylate MdANN2, resulting in reduced MdANN2 accumulation at the plasma membrane and weakened Ca^2+^ influx. These effects subsequently repress *CBF* and *COR* gene expression and compromise cellular tolerance to cold stress. Through this module, apple cells may fine‐tune the amplitude and duration of early Ca^2+^ signals, thereby balancing efficient defense activation with the maintenance of ion homeostasis. Together, these findings identify a previously unknown MdMPK6a/b–MdANN2 cold‐signaling module and provide potential targets for the genetic improvement of cold tolerance in perennial fruit crops.

**Figure 10:**
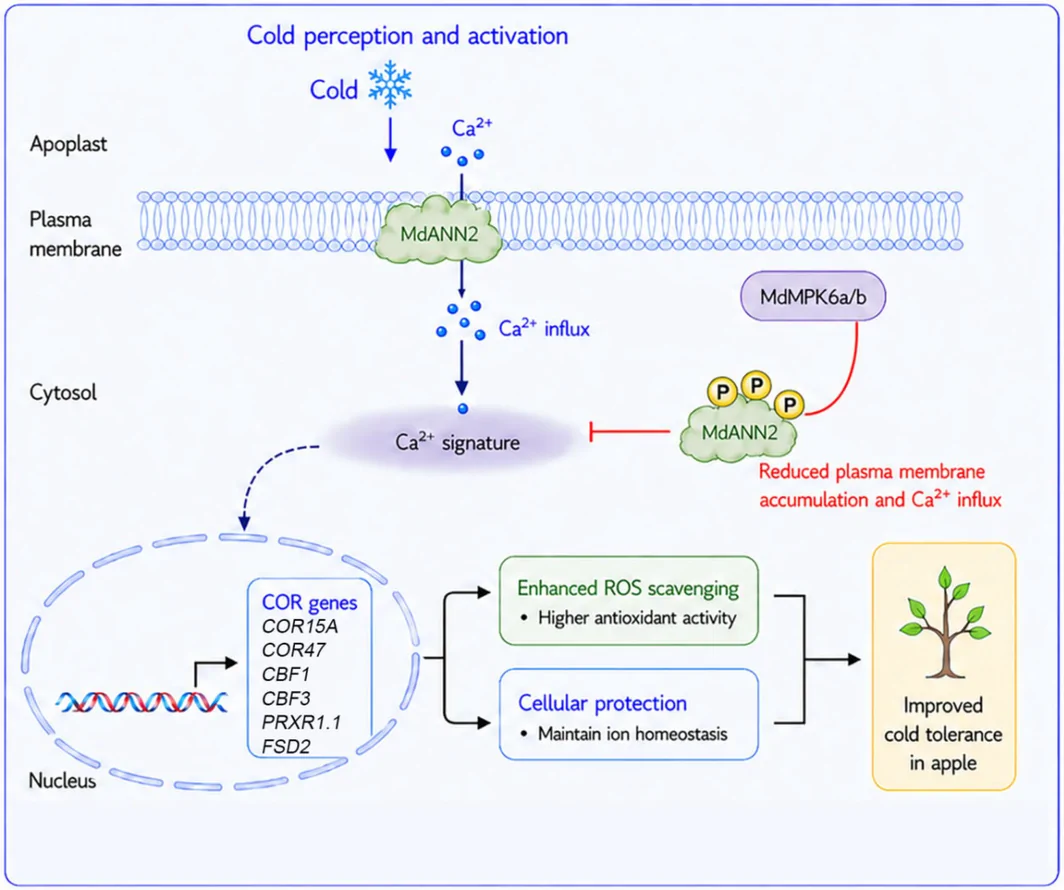
Working model of the MdMPK6a/b‐MdANN2 regulatory module in the apple cold response. Cold perception induces *MdANN2* expression and promotes its accumulation at the plasma membrane, where MdANN2 mediates Ca^2+^ influx and generates cytosolic Ca^2+^ signatures. These Ca^2+^ signals are transmitted to the nucleus to activate *CBF* transcription factors and induce the expression of *COR* (cold‐responsive) genes. Elevated *COR* gene expression enhances ROS scavenging and cellular protection, thereby improving apple cold tolerance. Cold stress also activates MdMPK6a and MdMPK6b, which phosphorylate MdANN2, reduce MdANN2 accumulation at the plasma membrane, suppress Ca^2+^ influx, and weaken the expression of downstream cold‐responsive genes. Thus, MdMPK6a and MdMPK6b act as negative regulators that fine‐tune MdANN2‐mediated cold‐induced Ca^2+^ signaling.

## MATERIALS AND METHODS

### Plant materials and growth conditions

The plant materials used in this study included apple “GL‐3” plants derived from “Royal Gala” (*M. domestica*) (Dai et al., 2013), “Orin” apple calli, and *N. benthamiana*. “GL‐3” plantlets were cultured on rooting medium for 40 days and then transplanted into pots containing a mixture of nutrient soil, vermiculite, and perlite (3:1:1, v/v/v). Plants were grown in a growth chamber under a long‐day photoperiod (16 h light/8 h dark). The rooting medium contained MS salts (4.43 g L^−1^), sucrose (30 g L^−1^), agar (8 g L^−1^), indole‐3‐butyric acid (IBA; 0.5 mg L^−1^), and indole‐3‐acetic acid (IAA; 0.5 mg L^−1^). *N. benthamiana* plants were grown in pots containing peat and vermiculite (1:1, v/v) under a 14 h light/10 h dark photoperiod. “Orin” calli were maintained in darkness at 24°C on MS medium supplemented with 1.5 mg L^−1^ 2,4‐dichlorophenoxyacetic acid (2,4‐D) and 0.4 mg L^−1^ 6‐benzylaminopurine (6‐BA) and subcultured every 20 days. Calli were used for *in vivo* phosphorylation assays. *N. benthamiana* plants were used for subcellular localization, bimolecular fluorescence complementation (BiFC), and dual‐luciferase assays.

### Genome‐wide identification and bioinformatic analysis of the MdANN gene family

For genome‐wide identification of MdANN family members, apple whole‐genome protein sequences (GDDH13) were downloaded from the Genome Database for Rosaceae (GDR; https://www.rosaceae.org/). The hidden Markov model file for the conserved annexin domain (PF00191) was downloaded from Pfam and used to search the apple proteome with HMMER 3.0. In parallel, *Arabidopsis* ANN protein sequences reported by Clark et al. (2001) and Cantero et al. (2006) were used as templates to search the GDDH13 apple proteome. The resulting MdANN candidates were further examined with SMART (https://smart.embl.de/smart/change_mode.cgi) and NCBI CDD (https://www.ncbi.nlm.nih.gov/Structure/bwrpsb/bwrpsb.cgi) to confirm the integrity of annexin domains.

For chromosomal localization and collinearity analysis, the GFF annotation file containing apple *ANN* gene position information (gene_models_20170612.gff3) was downloaded from GDR. Synteny among apple chromosomes was analyzed with MCScanX, and chromosomal locations and collinearity relationships were visualized with TBtools (Chen et al., 2023).

Phylogenetic trees were constructed in MEGA7 using the maximum‐likelihood method with 1000 bootstrap replicates, a JTT + G model, and partial deletion, and were visualized using iTOL (https://itol.embl.de/). The eight *Arabidopsis* ANN protein sequences were downloaded from TAIR (https://www.arabidopsis.org/), and 12 poplar ANN protein sequences were downloaded from JGI/Phytozome (https://phytozome‐next.jgi.doe.gov/) (Wei et al., 2022), as shown in Table S1.

Physicochemical properties of MdANN proteins, including molecular weight (MW), theoretical isoelectric point (pI), and instability index, were calculated using ExPASy ProtParam (https://web.expasy.org/protparam/) (Table S2). Three‐dimensional (3D) structures of MdANN proteins were predicted and visualized using SWISS‐MODEL (https://swissmodel.expasy.org/). Conserved motifs were identified with MEME (http://meme‐suite.org/), and motif architecture and gene structures were visualized using TBtools based on apple genome annotation files (Chen et al., 2023).

### Vector construction and genetic transformation

The coding sequence (CDS) of *MdANN2* was cloned into the pCambia2300‐GFP vector to generate the overexpression (OE) construct. The recombinant plasmid was transformed into *Agrobacterium rhizogenes* K599. Excised “GL‐3” leaves were immersed in a K599 suspension carrying the target plasmid and vacuum infiltrated at 0.09 MPa for 10 min. Leaves were then transferred to co‐cultivation medium containing MS salts (4.43 g L^−1^), sucrose (30 g L^−1^), agar (7 g L^−1^), thidiazuron (TDZ; 2 mg L^−1^), and naphthaleneacetic acid (NAA; 0.5 mg L^−1^) for 3 days. Subsequently, leaves were transferred to selection medium consisting of co‐cultivation medium supplemented with 250 mg L^−1^ cefotaxime sodium and 25 mg L^−1^ kanamycin. Resistant shoots were screened and confirmed by genomic PCR and RT‐qPCR.

For transient transformation assays, a vacuum‐infiltration method described previously was used with minor modifications (Zhang et al., 2024). pCambia2300‐GFP (control), pCambia2300‐GFP‐*MdMPK6a*, and pCambia2300‐GFP‐*MdMPK6b* were separately introduced into *MdANN2*‐OE or WT apple plants. After 48–72 h, three seedlings were randomly selected for RT–qPCR analysis to assess the efficiency of the transient transformation. Apple callus transformation was performed as described previously (Yang et al., 2021). Primers used in this study are listed in Table S3.

### Virus‐induced gene silencing (VIGS)

Virus‐induced gene silencing was performed as described previously with minor modifications (Dai et al., 2018). A 220 bp gene‐specific fragment of *MdANN2* and a 300 bp fragment corresponding to the conserved region shared by MdMPK6a and MdMPK6b were inserted separately into the pTRV2 vector. pTRV1, pTRV2, pTRV2‐*MdANN2*, and pTRV2‐*MdMPK6* were transformed into *Agrobacterium tumefaciens* GV3101. The combinations pTRV1 + pTRV2, pTRV1 + pTRV2‐*MdANN2*, and pTRV1 + pTRV2‐*MdMPK6* were vacuum‐infiltrated into apple leaves at 0.09 MPa for 10 min. After 2 weeks, RNA was extracted from three randomly selected seedlings, and the transcript levels of the target genes were quantified by RT–qPCR to evaluate the gene‐silencing efficiency.

### Low‐temperature treatments

After 40 days of growth under normal chamber conditions, apple plants of uniform size were selected for freezing treatment in a vertical cold‐light‐source growth chamber. The treatment was performed with slight modifications to previously described methods (Mei et al., 2023;). The temperature was decreased from 24 to 4°C at 4°C h^−1^ and then from 4 to −5°C at 2°C h^−1^; plants were maintained at −5°C for 3 h. After freezing treatment, all plants were recovered in darkness at 4°C for 12 h and then transferred to normal conditions at 24°C for 2 days. Three independent biological replicates were performed, with 18 plants per genotype per replicate. Survival rates were determined 2 weeks after recovery under normal conditions (16 h light/8 h dark, 24°C) and calculated as the number of surviving plants divided by the total number of plants multiplied by 100. For chilling treatment, plants or calli were incubated at 4°C for 8 h.

### Yeast two‐hybrid (Y2H) assay

The *MdANN2* coding sequence was cloned into pPR3‐N, and the coding sequences of *MdMPK6a/b* were cloned separately into pDHBI. The corresponding construct pairs were co‐transformed into the yeast strain NMY51 as described previously (Mao et al., 2021) and selected on SD/−Trp/−Leu medium. Positive colonies were transferred to SD/−Trp/−Leu/−His/−Ade medium and screened with X‐α‐Gal.

### Split‐luciferase complementation assay

The coding sequences of *MdMPK6a/b* were cloned separately into the pCambia1300‐nLUC vector, whereas the *MdANN2* coding sequence was cloned into pCambia1300‐cLUC. The recombinant plasmids were transformed into *Agrobacterium* GV3101 and co‐infiltrated into *N. benthamiana* leaves. Different construct combinations were infiltrated into separate regions of the same leaf. Luciferase activity was monitored within 48–60 h using a multispectral dynamic fluorescence microscopy imaging system (PlantView100, Guangzhou Biolight Biotechnology Co., Ltd., Guangzhou, China).

### Pull‐down assay

The coding sequences of *MdMPK6a/b* were cloned separately into pGEX‐4T‐1, and the *MdANN2* coding sequence was cloned into pET‐32a. Recombinant plasmids were transformed into *Escherichia coli* Rosetta2 (DE3) cells to express fusion proteins. Recombinant proteins were incubated *in vitro*, immunoprecipitated with His‐tag magnetic beads, and analyzed by immunoblotting with an anti‐GST antibody.

### Bimolecular fluorescence complementation (BiFC) assay

The *MdANN2* coding sequence was cloned into pSPYCE, and the coding sequences of *MdMPK6a/b* were cloned separately into pSPYNE. The recombinant constructs were transformed into *Agrobacterium* GV3101 and co‐infiltrated into *N. benthamiana* leaves. YFP fluorescence was observed 48–60 h after infiltration using a confocal laser scanning microscope (TCS‐SP8 SR; Leica, Wetzlar, Germany) following a previously described method (Kudla & Bock, 2016). The excitation wavelength for YFP was set to 514 nm.

### RT‐qPCR analysis

Total RNA was extracted using a plant RNA extraction kit (Foregene, Chengdu, China). cDNA was synthesized using the PrimeScript RT reagent kit (TaKaRa, Shiga, Japan). RT‐qPCR was performed on a LightCycler 96 system with the following reaction mixture: 10 μl SYBR Green I, 0.4 μl each of forward and reverse primers, 1 μl cDNA, and 8.2 μl deionized water. *MdEF‐1α* was used as the reference gene (Li et al., 2024), and relative expression levels were calculated using the 2^−ΔΔCt^ method (Livak & Schmittgen, 2001). Primers used in this study are listed in Table S3.

### DAB and NBT staining

Leaves were stained with 1 mg L^−1^ 3,3′‐diaminobenzidine (DAB; pH 3.8) or 0.5 mg L^−1^ nitroblue tetrazolium (NBT; pH 7.8). For DAB staining, leaves were vacuum infiltrated for 30 min and then incubated overnight at room temperature. For NBT staining, leaves were vacuum infiltrated and incubated for 2 h. Stained leaves were destained with 95% ethanol and photographed (Zhang et al., 2024).

### Measurement of H_2_O_2_ , $O_{2}^{-}$, antioxidant enzyme activities, and relative electrolyte leakage

H_2_O_2_ and $O_{2}^{-}$ contents and the activities of superoxide dismutase (SOD), peroxidase (POD), and catalase (CAT) were determined spectrophotometrically using commercial assay kits from Suzhou Comin Biotechnology Co., Ltd. (Suzhou, China) according to the manufacturer's instructions. Samples were ground in liquid nitrogen, homogenized in the appropriate extraction buffer, and centrifuged; the supernatant was collected for assays. After addition of the corresponding reaction reagents, absorbance was measured using a UV‐2600 spectrophotometer (Shimadzu, Tokyo, Japan). Relative electrolyte leakage (REL) was calculated as REL = [(D1 − D0)/(D2 − D0)] × 100% (Zhang et al., 2024).

### Subcellular localization

The *MdANN2* coding sequence was cloned into the pCambia2300‐GFP vector to generate *MdANN2*‐GFP, and the resulting construct was introduced into *Agrobacterium* GV3101. The nuclear marker NLS‐mCherry and the plasma membrane marker *AtCBL1*‐mCherry were used for co‐localization analysis, as previously described (Liu et al., 2026; Zhang et al., 2026). *Agrobacterium* strains carrying *MdANN2*‐GFP were co‐infiltrated into *N. benthamiana* leaves with NLS‐mCherry (nuclear marker) or AtCBL1‐mCherry (plasma‐membrane marker). pCambia2300‐GFP was used as the control. Fluorescence was observed 48–72 h after infiltration using a confocal laser scanning microscope (TCS‐SP8 SR; Leica, Germany). GFP and mCherry were excited at 488 and 552 nm, respectively.

To investigate the effect of MdMPK6a/b on the subcellular localization of MdANN2, subcellular localization assays were performed as described previously with minor modifications (Zhang et al., 2026). *Agrobacterium* GV3101 strains carrying MdANN2‐GFP, MdMPK6a‐HA, or MdMPK6b‐HA were infiltrated into *N. benthamiana* leaves. AtCBL1‐mCherry was used as a plasma‐membrane marker. 48–60 h after infiltration, MdANN2 localization was observed with a confocal laser scanning microscope (TCS‐SP8 SR; Leica, Germany), and fluorescence intensity was quantified using LAS‐X software. Furthermore, membrane proteins were extracted using a membrane protein extraction kit (Beyotime Biotechnology, Shanghai, China), and GFP and HA antibodies were used for western blot analysis.

### NMT assay

Net Ca^2+^ fluxes were measured using non‐invasive micro‐test technology (NMT150; Younger USA, Amherst, MA, USA) with minor modifications to previously described methods (Wang et al., 2021). The lower epidermis of each leaf was carefully removed, and leaves were equilibrated for 2 h in measuring solution containing 0.2 mM CaCl_2_, 1 mM KCl, and 0.2 mM MES (pH 5.8). Before each measurement, the Ca^2+^‐selective microelectrode was calibrated, and the samples were allowed to equilibrate until a stable baseline was achieved. Ca^2+^ fluxes were then continuously recorded before and immediately after cold treatment. Ca^2+^ fluxes were recorded at 24°C for approximately 5 min, after which the measuring solution was replaced with prechilled measuring solution at 4°C and fluxes were recorded for another 5 min. Positive and negative values indicate Ca^2+^ efflux and influx, respectively. Measurements were taken 5 μm from exposed mesophyll cells. EGTA and LaCl_3_ were added to final concentrations of 2 and 10 mM, respectively (Guo et al., 2025; Peng et al., 2024).

### Phosphorylation assay

*In vitro* phosphorylation assays were performed according to previously reported protocols (Song et al., 2023). Based on a previous report (Wang et al., 2022), the constitutively active mutants of MdMPK6a and MdMPK6b, designated CA‐MdMPK6a (D228G/E232A) and CA‐MdMPK6b (D225G/E229A), respectively, were generated by introducing two mutations into the conserved domains of each kinase. Recombinant proteins were expressed in *E. coli* Rosetta2 (DE3) cells. GST‐tagged CA‐MdMPK6a or CA‐MdMPK6b and His‐tagged MdANN2 were incubated at a 2:1 ratio in reaction buffer at 28°C for 2 h. The reaction buffer contained 25 mM Tris–HCl (pH 7.5), 10 mM MgCl_2_, 1 mM DTT, and 50 mM ATP. Reactions were terminated by adding protein loading buffer. Samples were separated on SDS‐PAGE gels containing 50 μM Phos‐tag acrylamide AAL‐107 (F4002; APExBIO, Houston, TX, USA), and MdANN2 was detected with an anti‐His monoclonal antibody (1:5000 dilution). Reactions treated with 1 μg calf intestinal alkaline phosphatase (CIAP) were used as controls.

For *in vivo* phosphorylation assays, *A. tumefaciens* EHA105 strains carrying MdANN2‐Flag and MdMPK6a/b‐HA were used to infiltrate apple calli. Total protein was extracted 3 days after infiltration using 600 μl of extraction buffer and incubated with 25 μl anti‐Flag magnetic beads. Samples were then separated by SDS‐PAGE, and the phosphorylation level of MdANN2 was detected using an anti‐phosphoserine/threonine antibody (PP2551; ECM Biosciences, USA) (Li et al., 2023).

### Statistical analysis

Statistical analyses were performed using SPSS v26.0 (IBM, Armonk, NY, USA), and data visualization was performed using GraphPad Prism v9.00. Error bars represent means ± SD from at least three biological replicates. Different letters indicate significant differences determined by one‐way analysis of variance followed by Duncan's multiple range test (*P* < 0.05).

## AUTHOR CONTRIBUTIONS

JW, JY and KM conceived and supervised the project. JW, MY, LJ and YL (Yehua Li) performed most of the experiments. YL (Yanghao Long), ZZ, JY and LQ contributed to the supplementary experiments. JW wrote the manuscript. FM and KM revised the manuscript.

## CONFLICT OF INTEREST

The authors declare no conflicts of interest.

## Supporting information


**Figure S1.** Bioinformatics analysis of the apple ANN gene family. (A) Distributions of MdANNs on nine chromosomes in apple. The graphical annotation indicates tandemly duplicated gene pairs. (B) Phylogenetic, gene structure, and conserved protein motif analyses of the apple ANN gene family. (C) The predicted 3D structure diagram of the ANN family proteins in apple.
**Figure S2.** Subcellular localization analysis of MdANN2. MdANN2‐GFP was co‐expressed with the mCherry‐tagged plasma membrane marker AtCBL1 and the NLS (nuclear localization signal) marker in *Nicotiana benthamiana* leaves to examine the subcellular localization of MdANN2. The images in the second row show magnified views of the regions indicated in the first row. Scale bars, 50 μm. GFP, green fluorescent protein; mCherry, red fluorescent protein.
**Figure S3.** Molecular identification of transgenic apple plants overexpressing MdANN2. (A) Genomic PCR identification of MdANN2 transgenic apple plants. WT, ‘GL‐3’ plants; positive, plasmid DNA of MdANN2‐GFP vectors; Negative, H_2_O. (b) MdANN2 transcripts in WT and transgenic lines. Error bars indicate the SD of three biological replicates. Different letters represent significant differences through one‐way ANOVA and Duncan's tests (*P* < 0.05).
**Figure S4.** Molecular identification of MdANN2‐silenced apple plants. Error bars indicate the SD of three biological replicates. Different letters represent significant differences through one‐way ANOVA and Duncan's test (*P* < 0.05).
**Figure S5.** Molecular identification of MdMPK6‐silenced apple plants. (A) The transcript levels of MdMPK6a. (B) The transcript levels of MdMPK6b. Error bars indicate the SD of three biological replicates. Different letters represent significant differences through one‐way ANOVA and Duncan's tests (*P* < 0.05).
**Figure S6.** Overexpression of MdMPK6a/b reduced cold tolerance in apple plants. (A) The transcript levels of MdMPK6a. (B) The transcript levels of MdMPK6b. (C) The phenotypic characteristics of apple plants under freezing treatment. (D) Survival rate. (E) Relative electrolyte leakage. (F) 3,3′‐diaminobenzidine (DAB) staining of leaves. (G) H_2_O_2_ content in apple leaves. (H) Nitro blue tetrazolium (NBT) staining of leaves. (I) $O_{2}^{-}$ content in apple leaves. (J) Superoxide dismutase (SOD) activity. (K) Peroxidase (POD) activity. (L) Catalase (CAT) activity. Error bars indicate the SD of three biological replicates. Different letters represent significant differences through one‐way ANOVA and Duncan's tests (*P* < 0.05).
**Figure S7.** Molecular validation of transient overexpression of MdMPK6a and MdMPK6b in stable MdANN2‐overexpressing plants. (A) The transcript levels of MdMPK6a. (B) The transcript levels of MdMPK6b. Error bars indicate the SD of three biological replicates. Different letters represent significant differences by one‐way ANOVA and Duncan's test (*P* < 0.05).
**Table S1.** ANN gene family members in *Arabidopsis thaliana* and *Populus trichocarpa*.
**Table S2.** Characteristics of the ANN family proteins in apple.
**Table S3.** Primer sequences used in this study.
**Table S4.** Candidate MdANN2‐interacting proteins identified by yeast two‐hybrid library screening.

## Data Availability

The original contributions presented in this study are included in the article/Supplementary Materials. Further inquiries can be directed to the corresponding author.
