# Supplementary material for: MdMPK6a/b‐mediated phosphorylation of MdANN2 restricts cytosolic Ca2+ influx and attenuates cold tolerance in apple
Source: Plant J. 2026 Oct 23;128(2):e71182. doi: 10.1111/tpj.71182 (PMC13691614; doi:10.1111/tpj.71182)
Supplement: Supplementary file 1 [file TPJ-128-0-s001.docx]

**Table S1. ANN gene family members in *Arabidopsis thaliana* and *Populus trichocarpa***

| *A. thaliana.* | | *P. trichocarpa* | |
| --- | --- | --- | --- |
| Gene | Gene accession No. | Gene | Gene accession No. |
| AtANN1 | AT1G35720 | PtANN1 | Potri.001G024800 |
| AtANN2 | AT5G65020 | PtANN2 | Potri.001G024900 |
| AtANN3 | AT2G38760 | PtANN3 | Potri.001G277500 |
| AtANN4 | AT2G38750 | PtANN4 | Potri.002G095600 |
| AtANN5 | AT1G68090 | PtANN5 | Potri.003G200700 |
| AtANN6 | AT5G10220 | PtANN6 | Potri.005G075900 |
| AtANN7 | AT5G10230 | PtANN7 | Potri.007G092500 |
| AtANN8 | AT5G12380 | PtANN8 | Potri.008G139500 |
|  |  | PtANN9 | Potri.010G101800 |
|  |  | PtANN10 | Potri.012G041900 |
|  |  | PtANN11 | Potri.013G055000 |
|  |  | PtANN12 | Potri.015G031800 |

**Table S2. characteristics of the ANN family proteins in apple.**

| Gene | Gene accession No. | Size（aa） | Molecular weight（KD） | Isoelectric point | Instability index |
| --- | --- | --- | --- | --- | --- |
| *MdANN1* | MD01G1029100 | 316 | 36.19 | 6.12 | 32.51 |
| *MdANN2* | MD02G1291000 | 313 | 35.68 | 6.88 | 35.12 |
| *MdANN3* | MD02G1291200 | 314 | 35.98 | 7.03 | 40.08 |
| *MdANN4* | MD02G1291300 | 353 | 39.87 | 7.61 | 41.21 |
| *MdANN5* | MD04G1000900 | 284 | 33.05 | 9.04 | 47.46 |
| *MdANN6* | MD05G1045300 | 318 | 36.38 | 6.51 | 38.48 |
| *MdANN7* | MD07G1035300 | 353 | 39.59 | 8.08 | 36.80 |
| *MdANN8* | MD07G1035400 | 315 | 36.12 | 6.35 | 43.67 |
| *MdANN9* | MD07G1036200 | 314 | 35.58 | 6.03 | 34.75 |
| *MdANN10* | MD07G1036300 | 359 | 40.60 | 6.65 | 39.90 |
| *MdANN11* | MD08G1187000 | 317 | 35.14 | 8.77 | 28.84 |
| *MdANN12* | MD12G1007300 | 321 | 36.73 | 7.17 | 45.82 |
| *MdANN13* | MD15G1367300 | 316 | 35.91 | 7.09 | 31.87 |
| *MdANN14* | MD15G1372900 | 317 | 35.28 | 9.14 | 33.77 |
| *MdANN15* | MD16G1013400 | 316 | 35.46 | 9.18 | 26.38 |

**Table S3. Primer sequences used in this study.**

| Annotation | Primer name | Primer sequence（5'-3') |
| --- | --- | --- |
| RT-qPCR | qMdANN1-F | TACGAGGGCTGAGGTTGACA |
|  | qMdANN1-R | AACCAGGGCCAGAAGCATTT |
|  | qMdANN2-F | ACATCGAGCTTCACACCTCC |
|  | qMdANN2-R | CGCGGAAGAGAGCCTTAGAG |
|  | qMdANN3-F | AGGTTCTTCGACTGGGCATC |
|  | qMdANN3-R | TTGCGTGGTCCAAATGAACG |
|  | qMdANN4-F | AAGACGAACTTTCCGGGGAC |
|  | qMdANN4-R | CGATGGCCTGAGTTGTCCTT |
|  | qMdANN5-F | GCAAAAAGGGATTGACCAGAGT |
|  | qMdANN5-R | CTCGACTAAAGAGACTCCGTGG |
|  | qMdANN6-F | ACAGAAATGCGGCACAGAGA |
|  | qMdANN6-R | GGCCACATGAGCCAAAACAG |
|  | qMdANN7-F | GCGCTTGTCCTCAGAGCTTA |
|  | qMdANN7-R | AATTGTCGCATCTGGGAGGG |
|  | qMdANN8-F | GCTCTCGGGACAGATGGC |
|  | qMdANN8-R | TGGTGTCTTGTTTGTTTGTTCGT |
|  | qMdANN9-F | TGTGGCCATGGAGAATTGGA |
|  | qMdANN9-R | ATCATGTCGATCTCAGCCCG |
|  | qMdANN10-F | TGTCCGCAAGCTTTTGGTTG |
|  | qMdANN10-R | GCCATGAGCTGAGTCTTGCT |
|  | qMdANN11-F | CGAGAGATGCACTGAGGACG |
|  | qMdANN11-R | GGGTAGGGAAACGGTTGAGG |
|  | qMdANN12-F | GAAGGGTTTGGGGACCGATG |
|  | qMdANN12-R | ATCGTTCAACGTCTTCCCGT |
|  | qMdANN13-F | AGCTCTTGGTTGCTCTTGTGA |
|  | qMdANN13-R | GGTGCCTCTTATCGACATCCTT |
|  | qMdANN14-F | AACGGAGTATTCCGCAGCTT |
|  | qMdANN14-R | TGCTTGCATACAACATCATAGCG |
|  | qMdANN15-F | GATGAGGATGCCCTCACTCG |
|  | qMdANN15-R | TCCTCGTAATCCCCGGAAGT |
|  | qMdCBF1-F | TCTTCCAACGCCAAGGACATTCAG |
|  | qMdCBF1-R | CGCCACAGGAGATCCACAAACC |
|  | qMdCBF3-F | TTGCCTCAACTTTGCCGACTCTG |
|  | qMdCBF3-R | GAAGCACCACAAGCCTCATCCG |
|  | qMdCOR47-F | AGGCTATCACAAGGAGGAGGACAC |
|  | qMdCOR47-R | GTCGGTTGGCTCTTCGTGGTAATG |
|  | qMdCOR15A-F | TCCTGTCTCTGTCAAACCGCTTTAC |
|  | qMdCOR15A-R | TCCTCTTCCCTCTCGCACTTCAC |
|  | qMdFSD2-F | AGCAGATAGAACTTAGGCGTATGA |
|  | qMdFSD2-R | TCTTATCCGTCAAGGTGGATACAAG |
|  | qMdPRXR1.1-F | ATGCTGATGTATGGATTTCAACGAT |
|  | qMdPRXR1.1-R | GGCAGCCTTCTCGGTTCTAA |
|  | qMdMPK6a-F | AGGTCACCGCCAAGTACAAGC |
|  | qMdMPK6a-R | TCTCCCTCAGCGTCCTCTTCG |
|  | qMdMPK6b-F | TGCGGTAAAGAAGATTGCGAATGC |
|  | qMdMPK6b-R | CTCTGCGGCGGAGGGATTATATC |
|  | qMdEF-1α-F | ATTCAAGTATGCCTGGGTGC |
|  | qMdEF-1α-R | CAGTCAGCCTGTGATGTTCC |
| Y2H | pDHBI-MdMPK6a-F | GCCATTACGGCCAGGCCTATGGAGGGAGGAGGGC |
|  | pDHBI-MdMPK6a-R | GGCCAAGATATACCATGGTCACTGTAGCTGATAC |
|  | pDHBI-MdMPK6b-F | GCCATTACGGCCAGGCCTATGGAGGGAGGAGGGC |
|  | pDHBI-MdMPK6b-R | GGCCAAGATATACCATGGTCACTGTCGCTGGTAC |
|  | pPR3-N-MdANN2-F | TTCCAGATTACGCTGGATCCATGGCAACCCTCACTG |
|  | pPR3-N-MdANN2-R | GATAAGCTTGATATCGAACTATTCTTCCTTCCCCAGC |
| Split-LUC | NLUC-MPK6b-F | GGACGAGCTCGGTACCATGGAGGGAGGAGGGC |
|  | NLUC-MPK6b-R | CCAGTCACTATGGTCGACCTGTCGCTGGTA |
|  | NLUC-MPK6a-F | GGACGAGCTCGGTACCATGGAGGGAGGAGGGC |
|  | NLUC-MPK6a-R | CCAGTCACTATGGTCGACCTGTAGCTGATAC |
|  | CLUC-MdANN2-F | TCCCGGGGCGGTACCATGGCAACCCTCACTG |
|  | CLUC-MdANN2-R | AAGCTCTGCAGGTCGACCTATTCTTCCTTCCCCAGC |
| BIFC | YNE-MdMPK6a-F | TGGCGCGCCACTAGTGGATCCATGGAGGGAGGAGGGCCC |
|  | YNE-MdMPK6a-R | AGCGGTACCCTCGAGGTCGACCTGTAGCTGATACTCGGGGTTAAA |
|  | YNE-MdMPK6b-F | TGGCGCGCCACTAGTGGATCCATGGAGGGAGGAGGGCGA |
|  | YNE-MdMPK6b-R | AGCGGTACCCTCGAGGTCGACCTGTCGCTGGTACTCGGGG |
|  | YCE-MdANN2-F | GCCTGGCGCGCCACTAGTGGATCCATGGCAACCCTCACTG |
|  | YCE-MdANN2-R | GGGAGCGGTACCCTCGAGGTCGACTTCCTTCCCCAGCAGA |
| Pull-down | 4T-MdMPK6a-F | CGTGGATCCCCGGAATTCATGGAGGGAGGAGGGC |
|  | 4T-MdMPK6a-R | ACGATGCGGCCGCTCGAGTCACTGTAGCTGATAC |
|  | 4T-MdMPK6b-F | CGTGGATCCCCGGAATTCATGGAGGGAGGAGGGC |
|  | 4T-MdMPK6b-R | ACGATGCGGCCGCTCGAGTCACTGTCGCTGGTAC |
|  | 32a-MdANN2-F | ATGGCTGATATCGGATCCATGGCAACCCTCACTG |
|  | 32a-MdANN2-R | GTGGTGGTGGTGCTCGAGTTCCTTCCCCAGCAGA |
| Phos | Flag-MdANN2-F | AGAACACGGGGGACTCTAGAATGGCAACCCTCACT |
|  | Flag-MdANN2-R | TGGTCCTTGTAATCGGTACCTTCCTTCCCCAGCAG |
|  | HA-MdMPK6a-F | GGGGACAAGTTTGTACAAAAAAGCAGGCTTCATGGAGGGAGGAGGGC |
|  | HA-MdMPK6a-R | GGGGACCACTTTGTACAAGAAAGCTGGGTTTCACTGTAGCTGATAC |
|  | HA-MdMPK6b-F | GGGGACAAGTTTGTACAAAAAAGCAGGCTTCATGGAGGGAGGAGGGC |
|  | HA-MdMPK6b-R | GGGGACCACTTTGTACAAGAAAGCTGGGTTTCACTGTCGCTGGTAC |
| Gene transfromation | OE-MdANN2-F | CGAGCTCGGTACCCGGGGATCCATGGCAACCCTCACTG |
|  | OE-MdANN2-R | CCTTGCTCACCATGGTGTCGACTTCCTTCCCCAGCAGA |
|  | TRV2-MdANN2-F | TGAGTAAGGTTACCGAATTCAGATCACTTCTCTTC |
|  | TRV2-MdANN2-R | GTGAGCTCGGTACCGGATCCGATCCAACGGTACAC |
|  | TRV2-MdMPK6-F | TGAGTAAGGTTACCGAATTCGGATAGGAAGCCATT |
|  | TRV2-MdMPK6-R | GTGAGCTCGGTACCGGATCCGGCTCATCACTGATG |
|  | OE-MdMPK6a-F | CGAGCTCGGTACCCGGGGATCCATGGAGGGAGGAGGGC |
|  | OE-MdMPK6a-R | CCTTGCTCACCATGGTGTCGACCTGTAGCTGATACTCG |
|  | OE-MdMPK6b-F | CGAGCTCGGTACCCGGGGATCCATGGAGGGAGGAGGGC |
|  | OE-MdMPK6b-R | CCTTGCTCACCATGGTGTCGACCTGTCGCTGGTACTCG |
| Subcellular localization | OE-MdANN2-F | CGAGCTCGGTACCCGGGGATCCATGGCAACCCTCACTG |
|  | OE-MdANN2-R | CCTTGCTCACCATGGTGTCGACTTCCTTCCCCAGCAGA |
|  | HA-MdMPK6a-F | GGGGACAAGTTTGTACAAAAAAGCAGGCTTCATGGAGGGAGGAGGGC |
|  | HA-MdMPK6a-R | GGGGACCACTTTGTACAAGAAAGCTGGGTTTCACTGTAGCTGATAC |
|  | HA-MdMPK6b-F | GGGGACAAGTTTGTACAAAAAAGCAGGCTTCATGGAGGGAGGAGGGC |
|  | HA-MdMPK6b-R | GGGGACCACTTTGTACAAGAAAGCTGGGTTTCACTGTCGCTGGTAC |

**Table S4. Candidate MdANN2-interacting proteins identified by yeast two-hybrid library screening.**

| **Gene ID** | **Description** |
| --- | --- |
| MD15G1147300 | mitogen-activated protein kinase 6 |
| MD10G1123300 | regulatory particle non-ATPase 10 |
| MD12G1260900 | Zinc-binding dehydrogenase family protein |
| MD17G1012600 | Ribosomal protein S21 family protein |
| MD14G1110700 | RNA binding Plectin/S10 domain-containing protein |
| MD00G1143000 | Ribosomal protein S5 domain 2-like superfamily protein |
| MD11G1136300 | Ribosomal protein L11 family protein |
| MD01G1043100 | cinnamyl alcohol dehydrogenase 9 |
| MD05G1290500 | double-stranded DNA-binding family protein |
| MD16G1202800 | double-stranded DNA-binding family protein |
| MD17G1174700 | DEA(D/H)-box RNA helicase family protein |
| MD15G1096500 | D-aminoacid aminotransferase-like PLP-dependent enzymes superfamily protein |

**Fig****ure.S1. Bioinformatics Analysis of the Apple *ANN* gene Family.** (a) Distributions of *MdANNs* on nine chromosomes in apple. The graphical annotation indicates tandemly duplicated gene pairs. (b) Phylogenetic, gene structure, and conserved protein motif analyses of the apple *ANN* gene family. (c) The predicted 3D structure diagram of the ANN family proteins in apple.

**Figure.S2. Subcellular localization analysis of MdANN2.** *MdANN2*-GFP was co-expressed with the mCherry-tagged plasma membrane marker AtCBL1 and the NLS (nuclear localization signal) marker in *Nicotiana benthamiana* leaves to examine the subcellular localization of MdANN2. The images in the second row show magnified views of the regions indicated in the first row. Scale bars, 50 μm. GFP, green fluorescent protein; mCherry, red fluorescent protein.

**Figure.S3. Molecular identification of transgenic apple plants overexpressing *MdANN2*.** (a) Genomic PCR identification of *MdANN2* transgenic apple plants. WT, 'GL-3' plants; positive, plasmid DNA of *MdANN2*-GFP vectors; Negative, H_2_O. (b) *MdANN2* transcripts in WT and transgenic lines. Error bars indicate the SD of three biological replicates. Different letters represent significant differences through one-way ANOVA and Duncan’s tests (p < 0.05).

**Figure.S4** **Molecular identification of *MdANN2*-silenced apple plants.** Error bars indicate the SD of three biological replicates. Different letters represent significant differences through one-way ANOVA and Duncan’s tests (p < 0.05).

**Figure.S5 Molecular identification of *MdMPK6*-silenced apple plants.** (a) The transcript levels of *MdMPK6a*. (b) The transcript levels of *MdMPK6b*. Error bars indicate the SD of three biological replicates. Different letters represent significant differences through one-way ANOVA and Duncan’s tests (p < 0.05).

**Figure.S6 Overexpression of *MdMPK6a/b* reduced cold tolerance in apple plants.** (a) The transcript levels of *MdMPK6a*. (b) The transcript levels of *MdMPK6b*. (c) The phenotypic characteristics of apple plants under freezing treatment. (d) Survival rate. (e) Relative electrolyte leakage. (f) 3,3'-diaminobenzidine (DAB) staining of leaves. (g) H_2_O_2_ content in apple leaves. (h) Nitro blue tetrazolium (NBT) staining of leaves. (i) O_2_^-^ content in apple leaves. (g) Superoxide dismutase (SOD) activity. (k) Peroxidase (POD) activity. (l) Catalase (CAT) activity. Error bars indicate the SD of three biological replicates. Different letters represent significant differences through one-way ANOVA and Duncan’s tests (p < 0.05).

**Figure.S7 Molecular validation of transient overexpression of *MdMPK6a* and *MdMPK6b* in stable *MdANN2*-overexpressing plants.** (a) The transcript levels of *MdMPK6a*. (b) The transcript levels of *MdMPK6b*. Error bars indicate the SD of three biological replicates. Different letters represent significant differences through one-way ANOVA and Duncan’s tests (p < 0.05).
